# Data on the gut and saliva microbiota from a cohort of atherosclerosis patients determined by 16S rRNA gene sequencing

**DOI:** 10.1016/j.dib.2018.05.032

**Published:** 2018-05-11

**Authors:** Gregory B. Gloor, Ruth Grace Wong, Emma Allen-Vercoe, Vincent Dinculescu, Michael Pignanelli, Chrysi Bogiatzi, Gregor Reid, J. David Spence

**Affiliations:** a Dept. Biochemistry, Schulich School of Medicine and Dentistry, Western University, London, Canada; b Dept. Molecular and Cell Biology, University of Guelph, Guelph, Canada; c Now at Department of Radiology, University of Calgary, Calgary, Canada; d Schulich School of Medicine and Dentistry, CIHR Summer Research Training Program, Canada; e Now at Dept. of Neurology, McMaster University, Hamilton, Canada; f Depts. of Urology and Microbiology, Schulich School of Medicine and Dentistry, Western University, and Lawson Research Institute, London, Canada; g Stroke Prevention & Atherosclerosis Research Centre, Robarts Research Institute, Western University, London, Canada

## Abstract

This work was conducted to characterize the 16S rRNA gene profile of a cohort of patients with traditional risk factors for developing atherosclerosis. The patients in the cohort were divided into two extremes; those predicted to develop extreme atherosclerosis who did not (Protected), and those predicted not to develop atherosclerosis who did (Unexplained). Bacterial DNA was isolated from stool and saliva and this was used to determine the V4 variable region of the 16S rRNA gene sequence composition of the samples in triplicate.

**Specifications Table**

**Table:** 

Subject area	*Biology*
More specific subject area	*Microbial Ecology*
Type of data	*Raw 16s rRNA gene sequencing reads from the V4 variable region*
How data was acquired	*Paired- end sequenced on an Illumina MiSeq using v3 chemistry*
Data format	*Raw reads*
Experimental factors	*Samples collected from consented volunteers*
Experimental features	*Very brief experimental description*
Data source location	*London, Canada. 43.01 −81.28*
Data accessibility	*Data deposited in the European Nucleotide Archive with accession number* PRJEB24929. Direct link is: https://www.ebi.ac.uk/ena/data/view/PRJEB24929

**Value of the data**

• The data was collected using the Earth Microbiome consortium V4 variable regions and should be comparable to other human microbiome studies that use the same primer sets.
• The data was collected in triplicate and can be used as a reference dataset to characterize technical variation in human microbiome studies.
• The data are from a cohort of patients with extreme phenotypes of atherosclerosis and can be used in meta-analyses of this condition.
• The data are from two different body sites, and can be used to compare the microbiota in different human niches.

## 1 Data

The data are PCR-amplified V4 variable regions that were paired-end sequenced on an Illumina MiSeq. The raw fastq file was downloaded and demultiplexed based upon identity to combinatorial barcodes [1]. The reads and associated metadata are deposited in the European Nucleotide archive (https://www.ebi.ac.uk/ena) with the accession number PRJEB24929, and can be accessed with the link: https://www.ebi.ac.uk/ena/data/search?query=PRJEB24929.

## 2 Experimental design, materials and methods

The study and participant cohorts are described in ref. [2]. In brief, patients were phenotyped by carotid artery plaque by JDS, and metadata collected. The metadata and carotid artery plaque were used to partition patients into groups. Protected patients had very high traditional risk scores but very low plaque burden and Unexplained patients had very low traditional risk scores but a very high plaque burden. Participants gave written informed consent to an ethics protocol approved by the Western University Health Sciences Research Ethics board (approval number 12107E).

Fresh fecal and saliva samples from 20 volunteers in each group that were the most extreme in phenotype were frozen on dry ice within 1 h of sampling. Samples were thawed and purified using the standardized methods described [3]. The protocol uses several off-the-shelf components. Briefly 200 ul of sample was diluted in 300 ul of SLX buffer from the Omega Bio-Tek E.Z.N.A.® Stool DNA kit (Norcross, Georgia) and 10 ul of 20 mg/ml proteinase K and 200 mg of glass beads were added subsequently. Samples were beat vigorously for 3 min at room temperature and then incubated at 70 °C for 10 min, 95 °C for 5 min and 4 °C for at least 2 min. The E.Z.N.A. protocol was followed exactly until the inhibitor removal step. At this point the samples were transferred into Maxwell®16 DNA Purification Kit cartridges from Promega (Madison, Wisconsin), and the extraction carried out according to the instructions in this kit without modification.

One to five ng of purified DNA was used as input for PCR amplification using the Earth Microbiome primers for the V4 variable region of the 16S rRNA gene. The exact protocol, primers, barcodes and standard operating procedures (SOP) are permanently archived at: https://github.com/ggloor/miseq_bin/releases/tag/v1.0. In brief, the primers used were: GTGCCAGCMGCCGCGGTAA, and GGACTACHVGGGTWTCTAAT, with 8-mer inline barcodes according to a published protocol [1]. PCR amplification was carried out with 1–5 ng of input DNA using the protocol in the SOP for 25 cycles. The amount of DNA in the tubes following the PCR reactions was quantitated using QuBit dsDNA, and pooled at approximately equimolar amounts as in the SOP. A second amplification for 10 cycles was conducted to attach the Illumina adaptor sequences and the pooled samples were run of a 2 × 300 cycle MiSeq run using version 3 chemistry at the London Regional Genomic Center. Samples were amplified, purified, and sequenced in triplicate with unique combinatorial barcodes.

Samples were processed for analysis using the software released in the archived pipeline, using the barcode and sample information in the metadata table attached to the ENA archived data.

The actual reads deposited in the ENA were demultiplexed with the Perl script ‘demultiplex_dada2.pl’ archived at https://github.com/ggloor/miseq_bin. This script demultiplexes inline combinatorial barcoded paired-end fastq sequences into forward and reverse files, one per sample per direction, with the primers and barcodes removed. Samples were named according to the convention given in the MiXS file attached to the archived DNA reads.

**Fig. 1:**
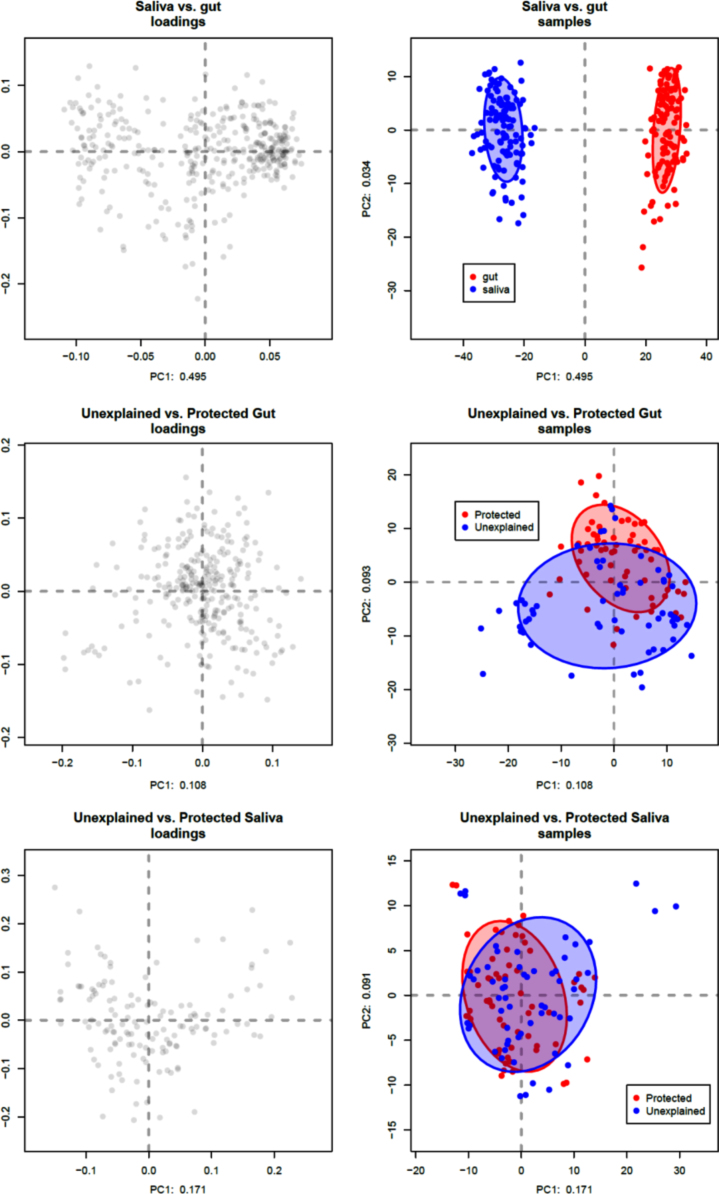
Loadings plots and PCA plots of the data. Loadings plots (left side) show the contribution of each OTU to the observed distances shown in the PCA plots (right side). Groups are colored red and blue as in the legend. The circles encompass the 75% confidence interval. The saliva vs. gut comparison accounts for nearly 50% of the variance in the data, and the two sample types do not overlap. The Unexplained vs. Protective atherosclerosis comparison accounts for no more than 17% of the variance in the data and the microbiota compositions overlap in both the gut and saliva comparisons. Additional plots are shown in a supplement. (For interpretation of the references to color in this figure legend, the reader is referred to the web version of this article.)

The actual table of counts is appended as ‘td_OTU_tag_mapped.txt’, and the minimal metadata file appended as ‘category. txt’. The read table was processed using a compositional data analysis paradigm [4], [5], [6], [7] exactly as in biplot_script.R to produce Fig. 1.

In brief, the operational taxonomic units (OTUs) were filtered to remove OTUs that contribute less than the median amount of variance of all OTUs to the dataset. In the entire dataset, this reduced the number of OTUs from 984 to 492; low variance OTUs are not informative by definition. A secondary filter was applied to remove all OTUs with a relative abundance less than 0.5%, reducing the OTU table from 492 OTUs to 363. Filtering in this way can be done without a loss of information on the composition when using a compositional data analysis paradigm [@bian:2017]. Data were subset to only gut, or only saliva and the operations repeated. The gut only samples had 289 OTUs and the saliva only samples had 136 OTUs after filtering. Zero count reads were imputed with the “CZM” function of the zCompositions R package [8] prior to centered log-ratio (clr) transformation [9]. Data transformed by the clr are most appropriate when analyzing high throughput sequencing data of this type [4], and the values represent linear differences in the ratio abundances. The clr transformed data are appropriate for analyses based on linear differences, and were used as input for Principle Component Analysis (PCA) [10], [11]. Fig. 1 shows the PCA plots where the saliva vs. gut samples were compared, and the plots of the Unexplained vs. Protective atherosclerosis samples for both gut and saliva.
