# Supplementary material for: Data on the gut and saliva microbiota from a cohort of atherosclerosis patients determined by 16S rRNA gene sequencing
Source: Data Brief. 2018 May 11;19:481–5. doi: 10.1016/j.dib.2018.05.032 (PMC5997836; doi:10.1016/j.dib.2018.05.032)

Data on the gut and saliva microbiota from a cohort of atherosclerosis patients determined by 16S rRNA gene sequencing

Data in Brief

Gregory Gloor, Ruth Wong, Emma Allen-Vercoe, Vincent Dinculescu, Michael Pignanelli, Chrysi Bogiatzi, Gregor Reid, J. David Spence,

Executive Summary

After attempting various distance based approaches (UniFrac) and variance based approaches (ALDEx, compositional data analysis), and by categorizing sequences by Operational Taxonomic Units or Individual Sequence Units, we found no difference between the Protected and Unexplained groups, with an absolute residual score greater than 2. Additionally, there is no consistent difference between males and females. The non significant effects found are not consistent between males and females, or the extreme (residual score > 2) and intermediate (residual scores between 1 and 2) groups. We also explored secondary outcomes and descriptors in the metadata, and nothing in the microbiota correlated or was associated with these. In conclusion, no consistent taxonomic group that can be identified that correlates with the clinical features of the patients.

Methods

DNA was amplified by PCR using the Earth Microbiome V4 primer set (Caparoso, 2012), with the addition of combinatorial in-line barcodes so that all the samples could be sequenced in the same sequencing run (Gloor, 2010). The DNA was sequenced on the Illumina MiSeq platform with paired end 220 nucleotide reads, producing sixteen million reads in total.

Reads were overlapped and filtered such that there is no more than 1 error per overlap with USEARCH (Edgar, 2010), clustered into Operational Taxonomic Units (OTUs) using UCLUST (Edgar, 2010), and annotated with the SILVA database (Quast, 2013) using mothur (Schloss, 2009), producing a table of counts per operational taxonomic unit per sample. Eight million of the reads were successfully overlapped and annotated into 299 OTUs. Differential abundance was analyzed using ALDEx2 (Fernandes, 2014). An Individual Sequence Unit (ISU) based approach was also used through the DADA2 software package (Callahan, 2015). A generalized workflow for processing 16S rRNA gene sequencing reads is available at https://github.com/ggloor/miseq_bin. The workflow for the 16S rRNA gene tag experiment analysis from the count table stage is on GitHub: https://github.com/ruthgrace/spence_atherosclerosis_16S.

Callahan, Benjamin J., et al. "DADA2: High resolution sample inference from amplicon data." bioRxiv (2015): 024034.

Caporaso, J. Gregory, et al. "Ultra-high-throughput microbial community analysis on the Illumina HiSeq and

MiSeq platforms." The ISME journal 6.8 (2012): 1621-1624.

Edgar, Robert C. "Search and clustering orders of magnitude faster than BLAST." Bioinformatics 26.19 (2010): 2460-2461.

Fernandes, Andrew D., et al. "Unifying the analysis of high-throughput sequencing datasets: characterizing RNA-seq, 16S rRNA gene sequencing and selective growth experiments by compositional data analysis."Microbiome 2.1 (2014): 1.

Gloor, Gregory B., et al. "Microbiome profiling by illumina sequencing of combinatorial sequence-tagged PCR products." PloS one 5.10 (2010): e15406.

Quast, Christian, et al. "The SILVA ribosomal RNA gene database project: improved data processing and web-based tools." Nucleic acids research41.D1 (2013): D590-D596.

Schloss, Patrick D., et al. "Introducing mothur: open-source, platform-independent, community-supported software for describing and comparing microbial communities." Applied and environmental microbiology 75.23 (2009): 7537-7541.

Data

There were 205 patients in total in this set. For this analysis we ignored the patients with a residual score between -1 and 1, and only looked at the high residual scores. We refer to patients with residual scores between 1 and 2 or -1 and -2 as ‘intermediate’, and patients with residual scores greater than 2 or less than -2 as ‘extreme’. The intermediate and extreme groups are further split by male and female sex, resulting in four groups total (intermediate male, intermediate female, extreme male, extreme female), which have been analyzed separately such that the results can be cross-referenced.

The metadata provided were: total plaque area (mm2), age, sex (male/female), smoking status (yes/no/quit), pack years, systolic blood pressure, diastolic blood pressure, cholesterol, triglyceride, HDL, LDL, standard predicted value, and residual score.

For the Operational Taxonomic Unit-based approach, OTUs were filtered to exclude any OTUs that did not exceed 1% abundance in any sample. For the Individual Sequence Unit-based approach, ISUs were filtered to exclude any ISUs that did not exceed 0.1% abundance.

Sample contents

OTU Level

The most highly abundant OTUs were annotated as follows, ranging from an average of 6.5% abundance to 1.7% abundance across all 205 samples.

Most abundant	Genus annotation	jackknifing confidence score
1	Bacteroides	100
2	Bacteroides	100
3	Pseudobutyrivibrio	95
4	Escherichia-Shigella	100
5	Faecalibacterium	100
6	Blautia	96
7	Akkermansia	100
8	Streptococcus	100
9	Bacteroides	100
10	Subdoligranulum	100

Genus level

The following are dendrogram barplots for various conditions, with OTUs aggregated at the genus level. None of the plots separated the sample by group at the OTU, genus, or family level.

Here are the dendrogram barplots for the patients of male sex in the extreme (residuals > 2 or<2) group. The OTUs are aggregated at the genus level.

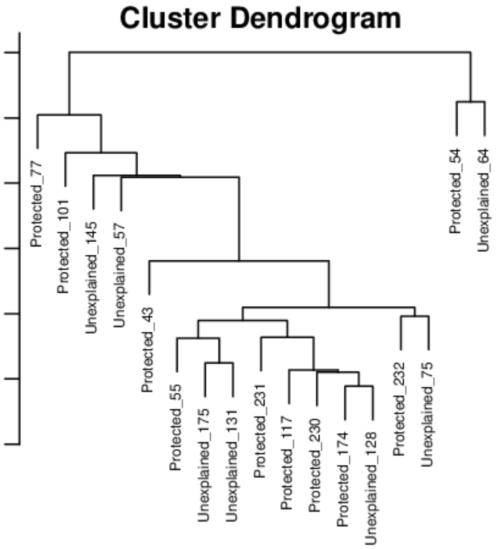

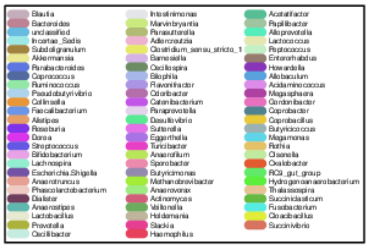

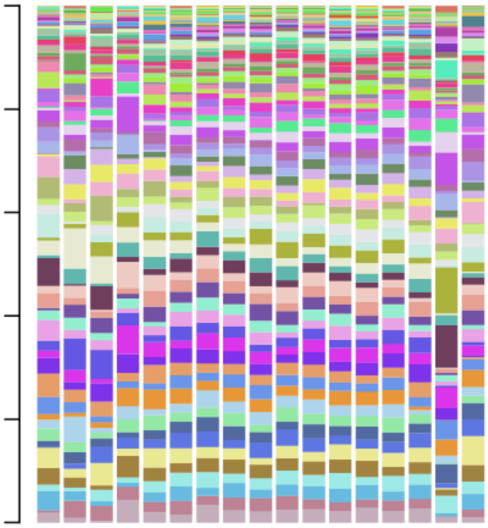

Here are the dendrogram barplots for the patients of male sex in the intermediate (residuals between 1 and 2 or -1 and -2) group. The OTUs are aggregated at the genus level.

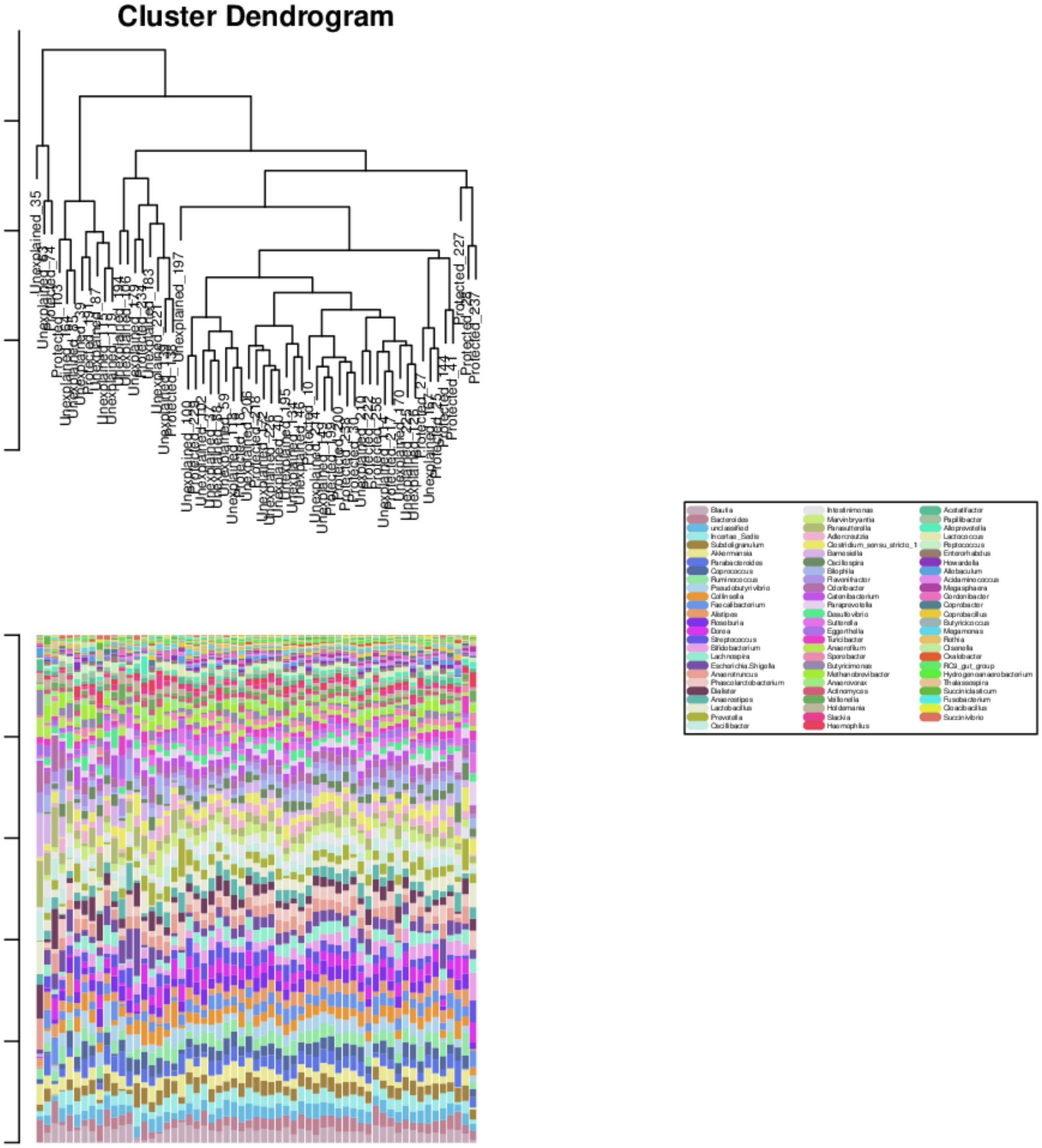

Here are the dendrogram barplots for the patients of female sex in the intermediate (residuals between 1 and 2 or -1 and -2) group. The OTUs are aggregated at the genus level.

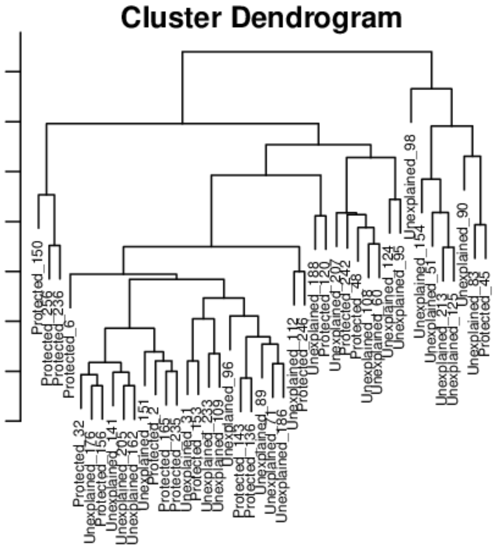

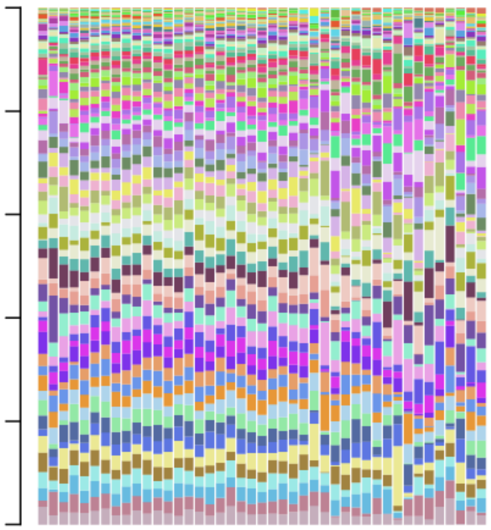

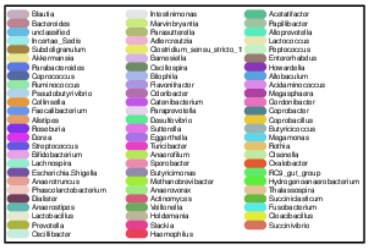

Differential Abundance

No taxa were found to be significantly differentially abundant in either the OTU based approach or the ISU based approach. Furthermore, the taxa found to be the most differentially abundant did not correspond between the male, female, intermediate, and extreme groups.

Operational Taxonomic Unit based approach

Difference between vs. difference within protected and unexplained conditions for each OTU, for patients of the male sex in the extreme (residuals > 2 and < -2) group

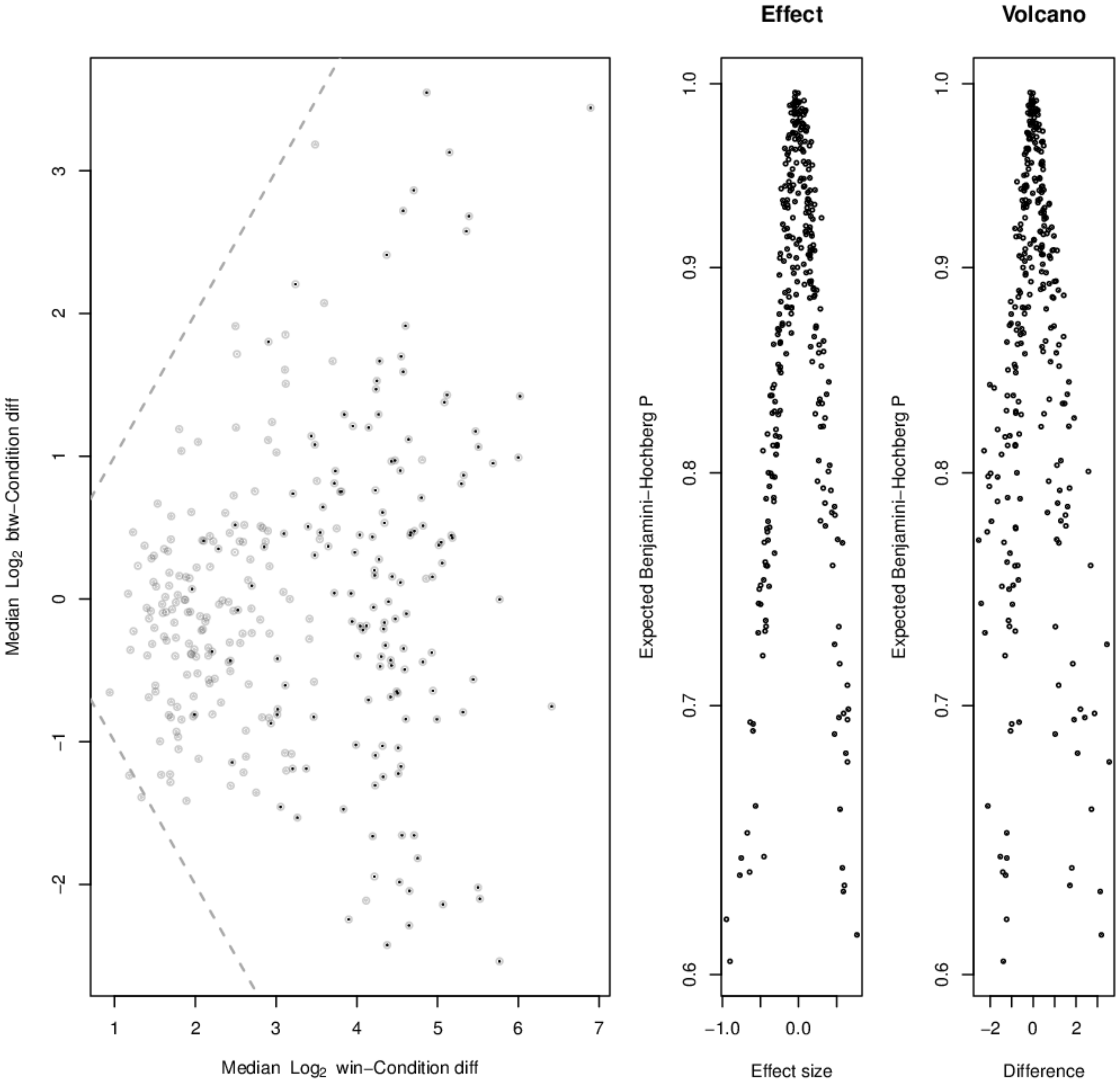

Difference between vs. difference within protected and unexplained conditions for each OTU, for patients of the female sex in the extreme (residuals > 2 and < -2) group

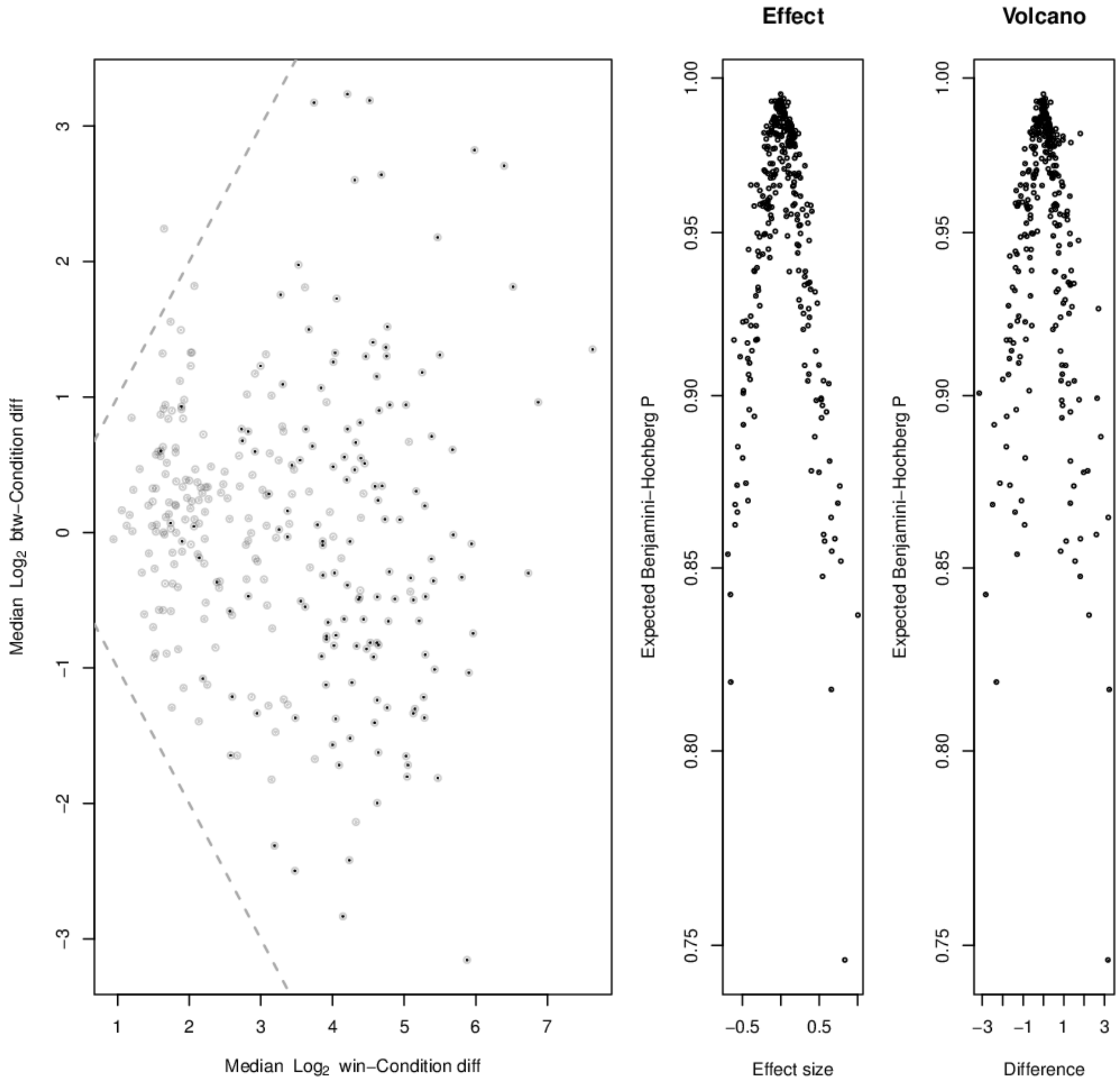

Difference between vs. difference within protected and unexplained conditions for each OTU, for patients of the male sex in the intermediate (residuals between 1 and 2 or -1 and -2) group

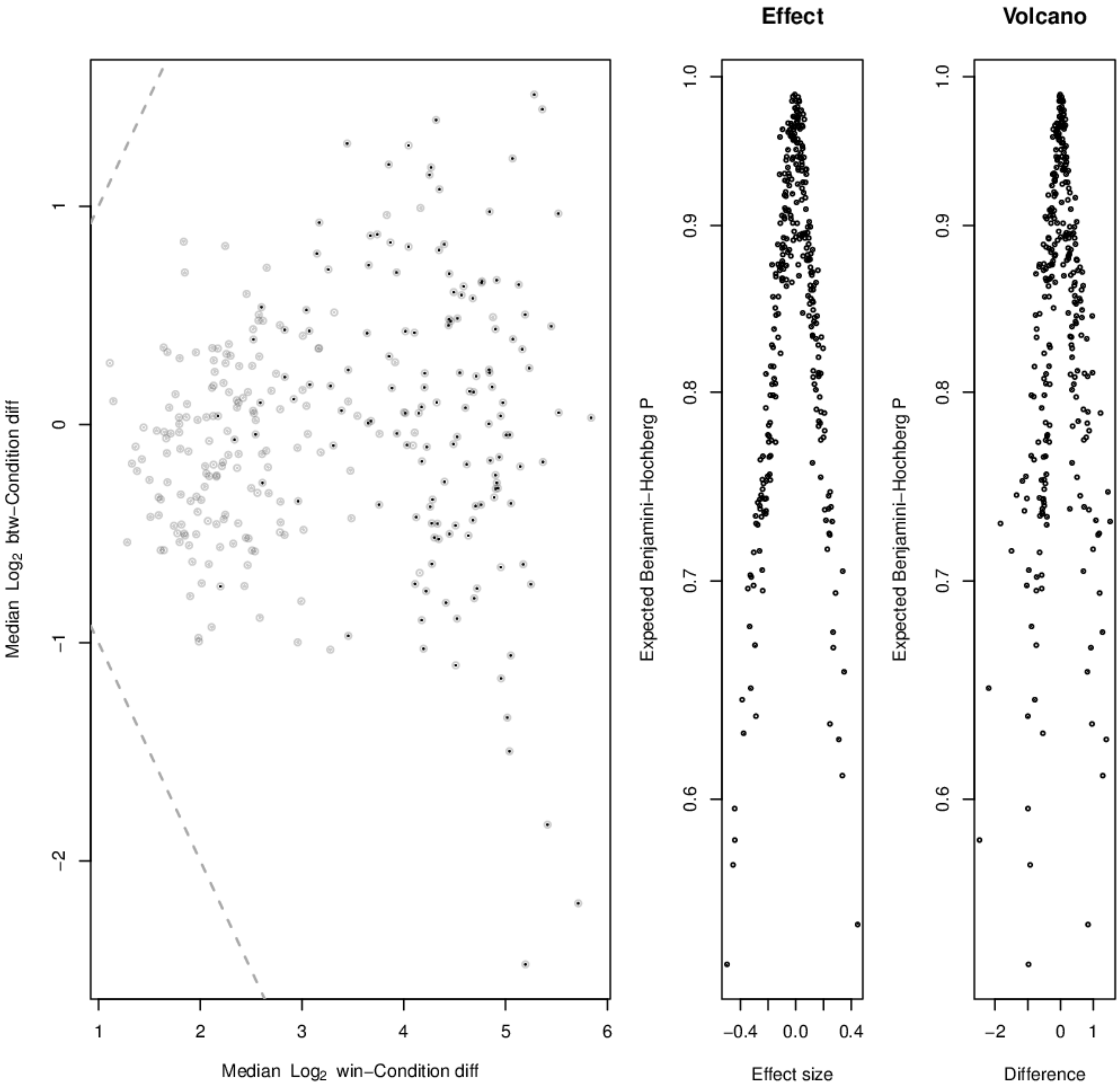

Difference between vs. difference within protected and unexplained conditions for each OTU, for patients of the female sex in the intermediate (residuals between 1 and 2 or -1 and -2) group

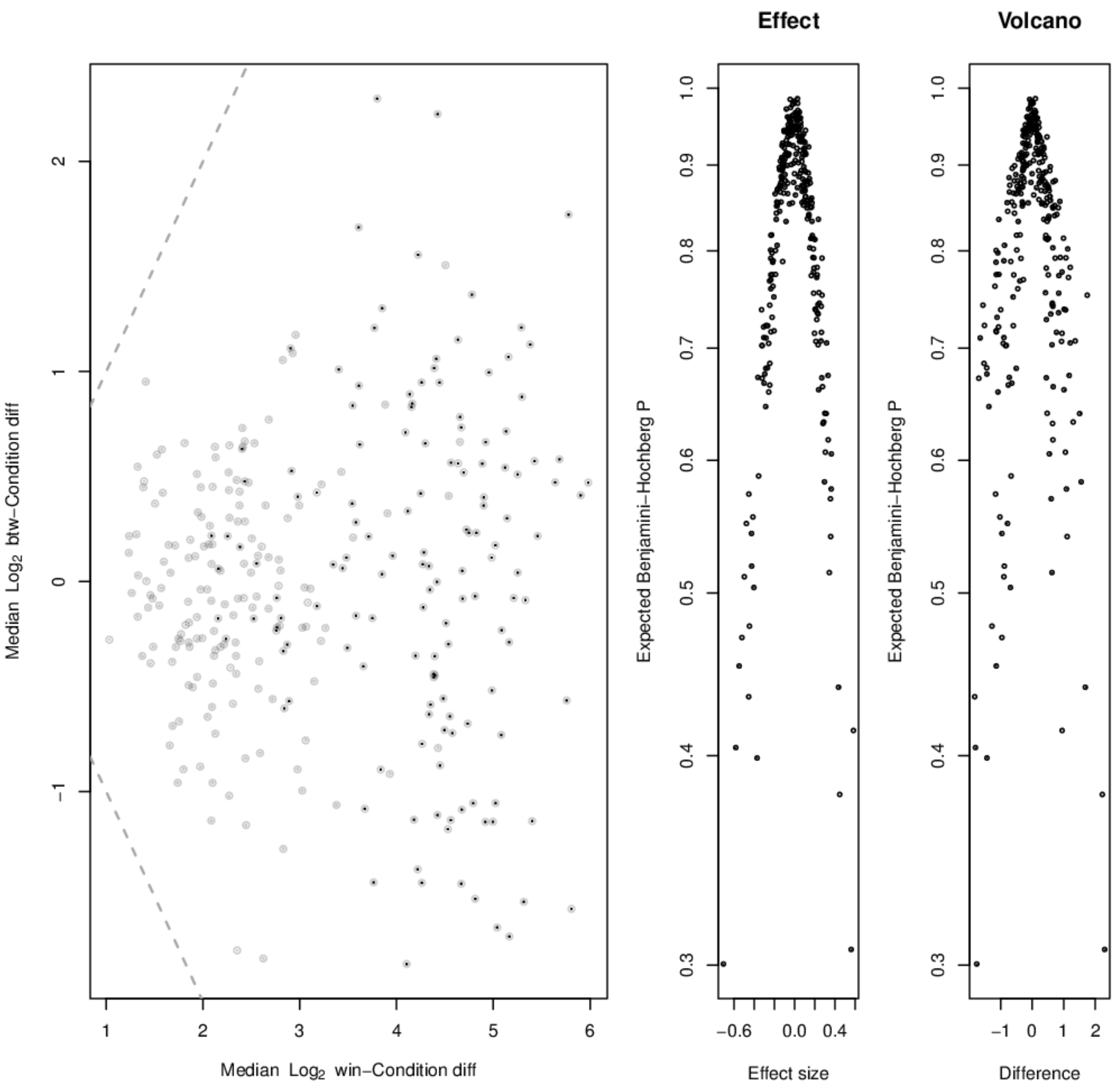

Venn diagram of all OTUs with an effect size of greater than 0.4 (relatively abundant in the Unexplained condition)

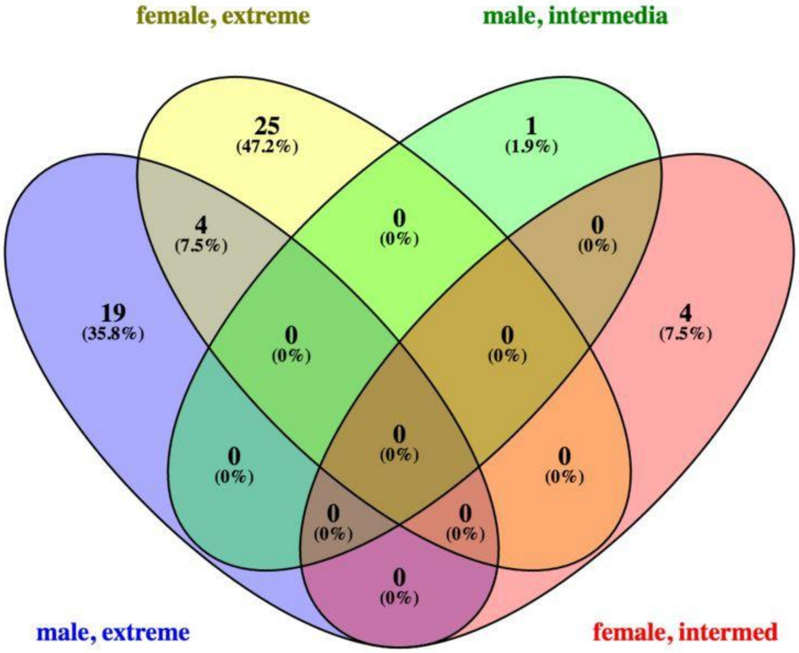

Venn diagram of all OTUs with an effect size of less than -0.4 (relatively abundant in the Protected condition)

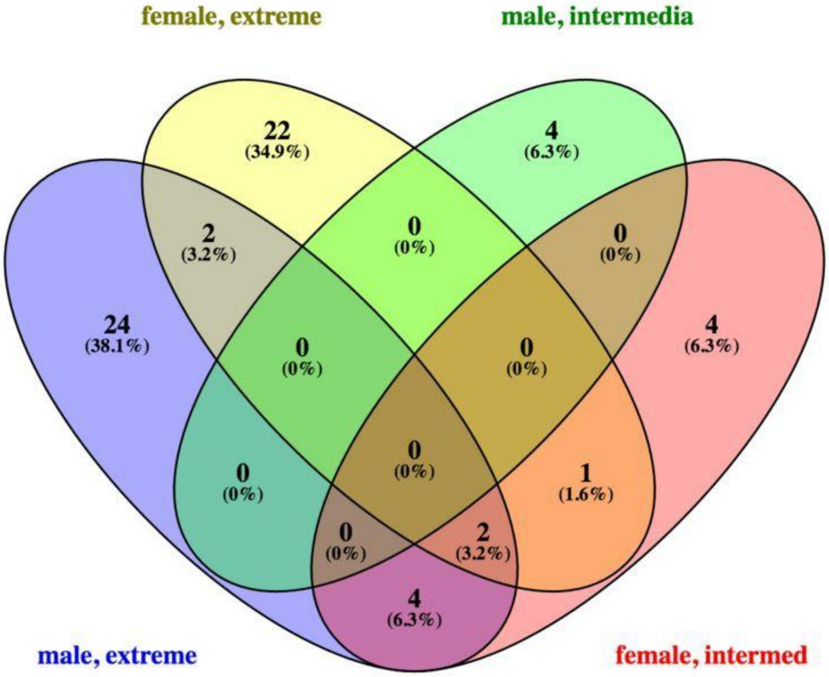

Individual Sequence Unit based approach

Difference between vs. difference within protected and unexplained conditions for each ISU, for patients of the male sex in the extreme (residuals > 2 and < -2) group

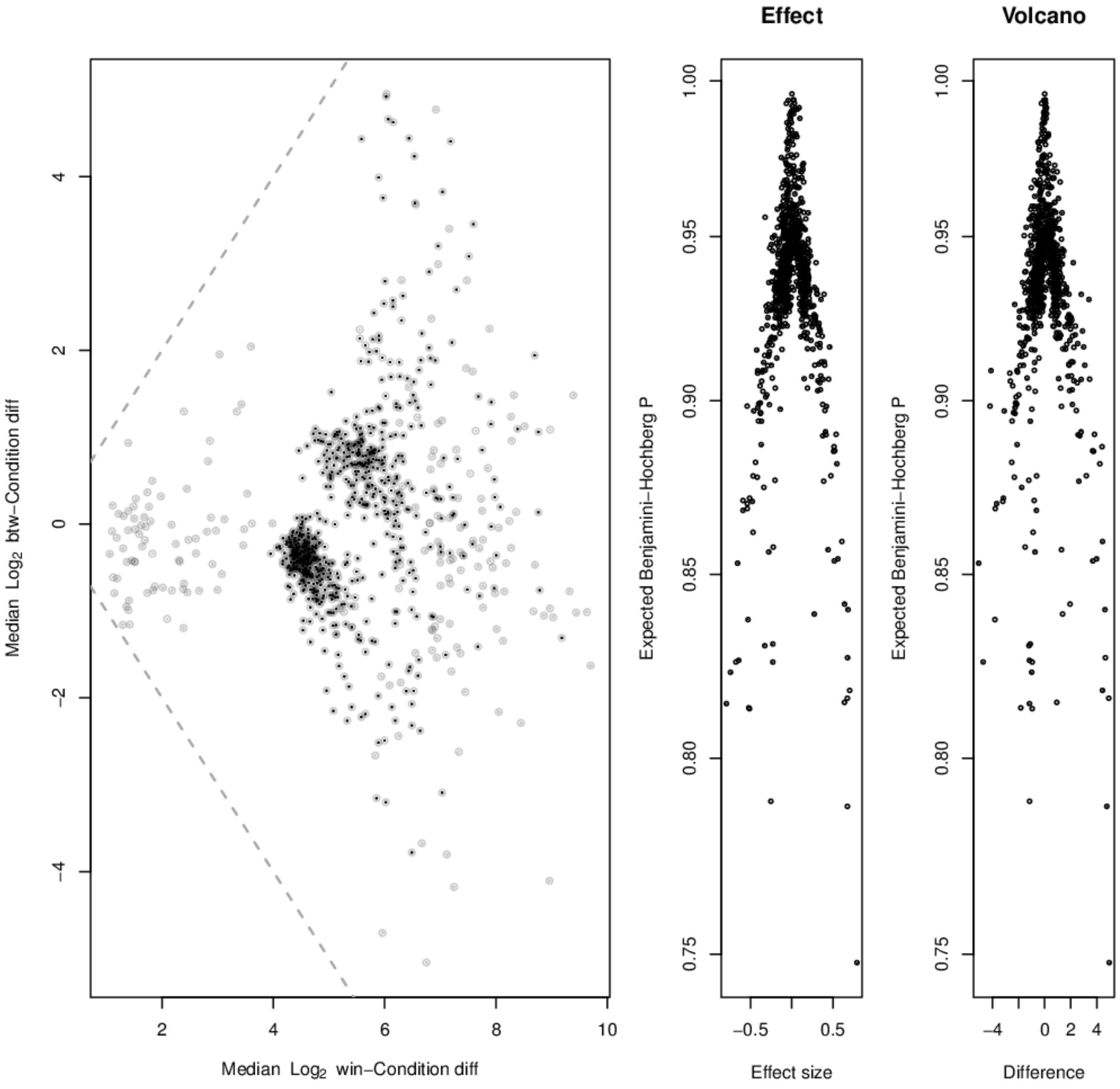

Difference between vs. difference within protected and unexplained conditions for each ISU, for patients of the female sex in the extreme (residuals > 2 and < -2) group

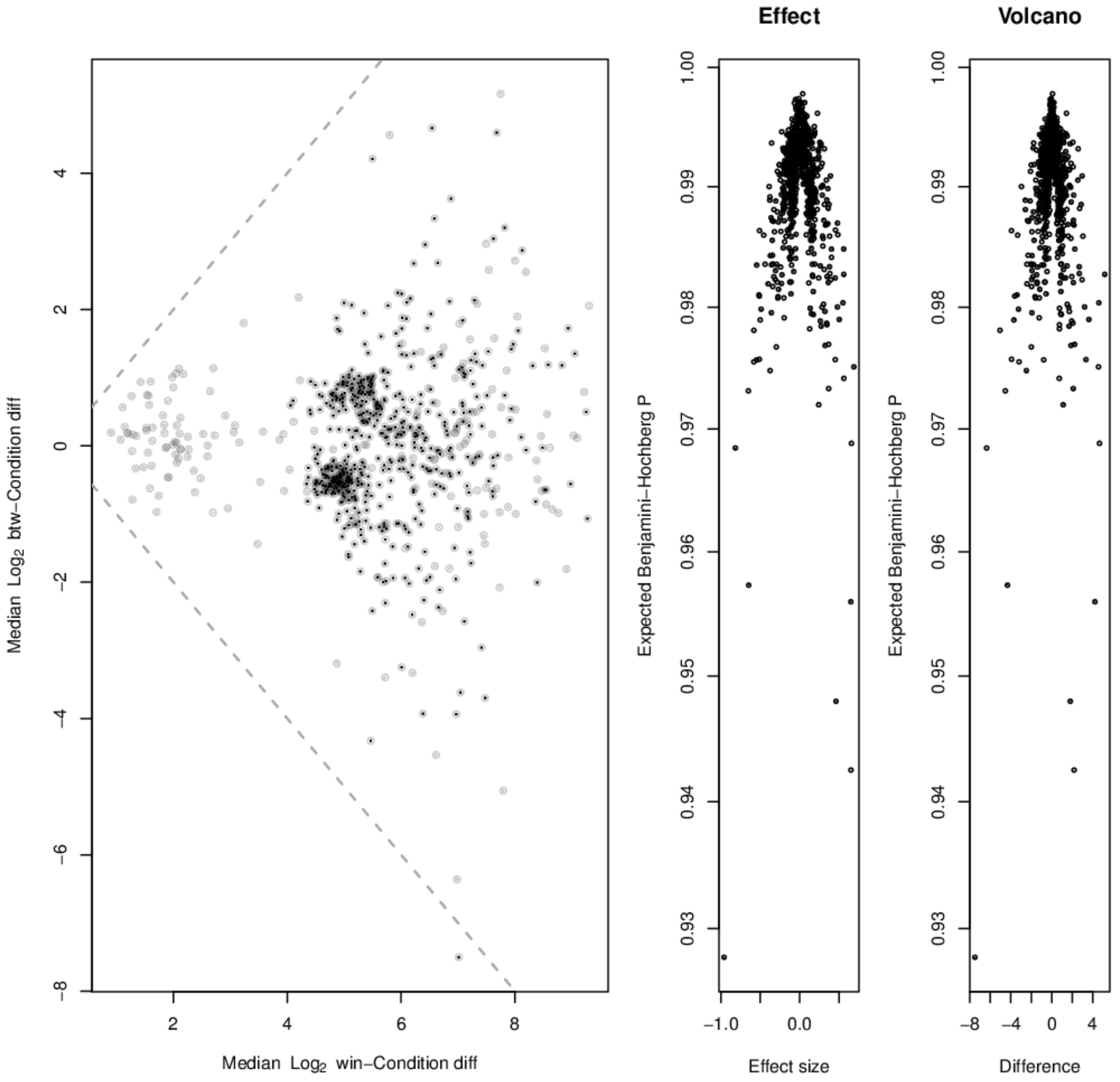

Difference between vs. difference within protected and unexplained conditions for each ISU, for patients of the male sex in the intermediate (residuals between 1 and 2 or -1 and -2) group

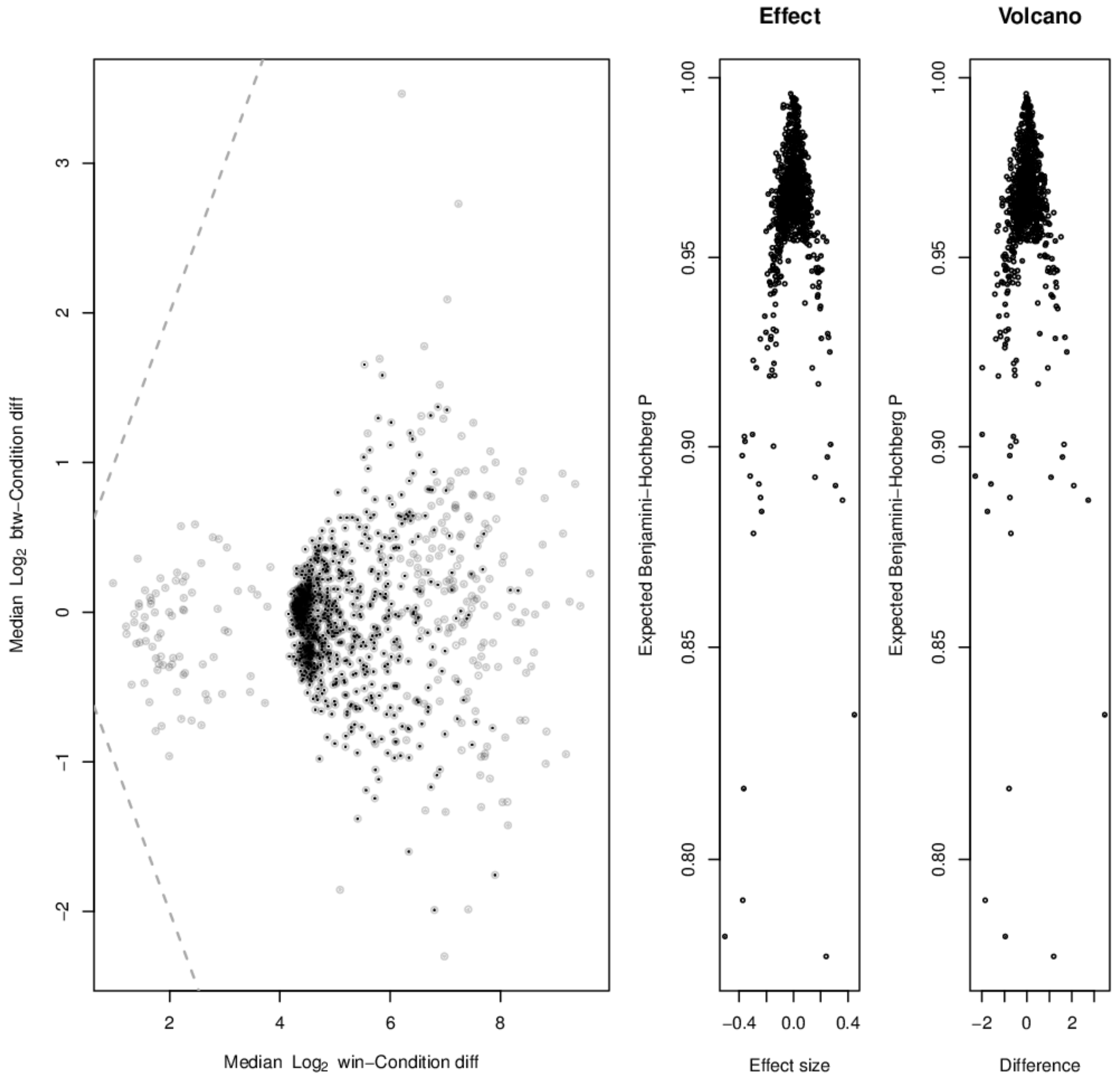

Difference between vs. difference within protected and unexplained conditions for each ISU, for patients of the female sex in the intermediate (residuals between 1 and 2 or -1 and -2) group

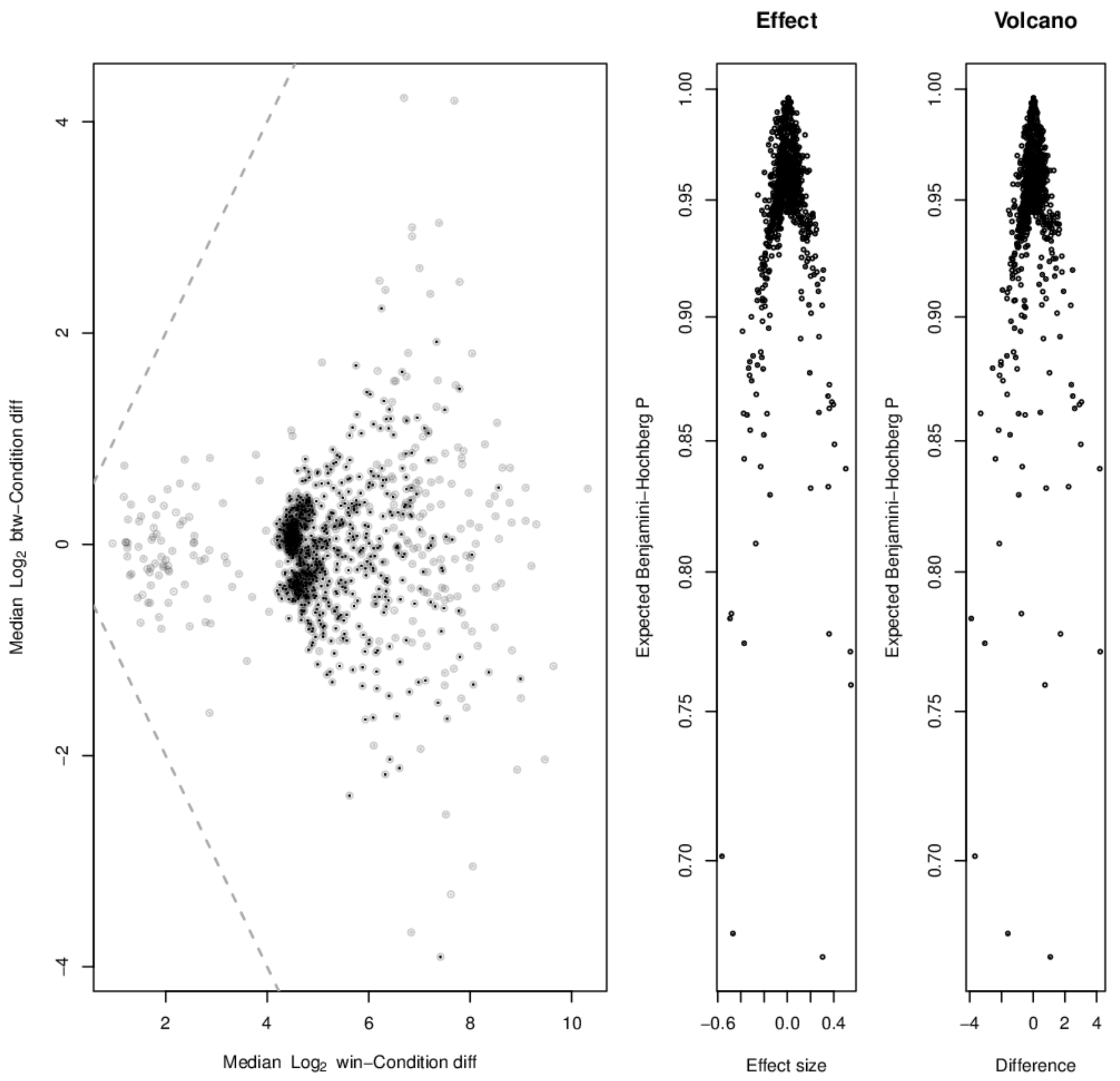

There appear to be two clusters of points in the rare counts for the extreme conditions, circled in red below.

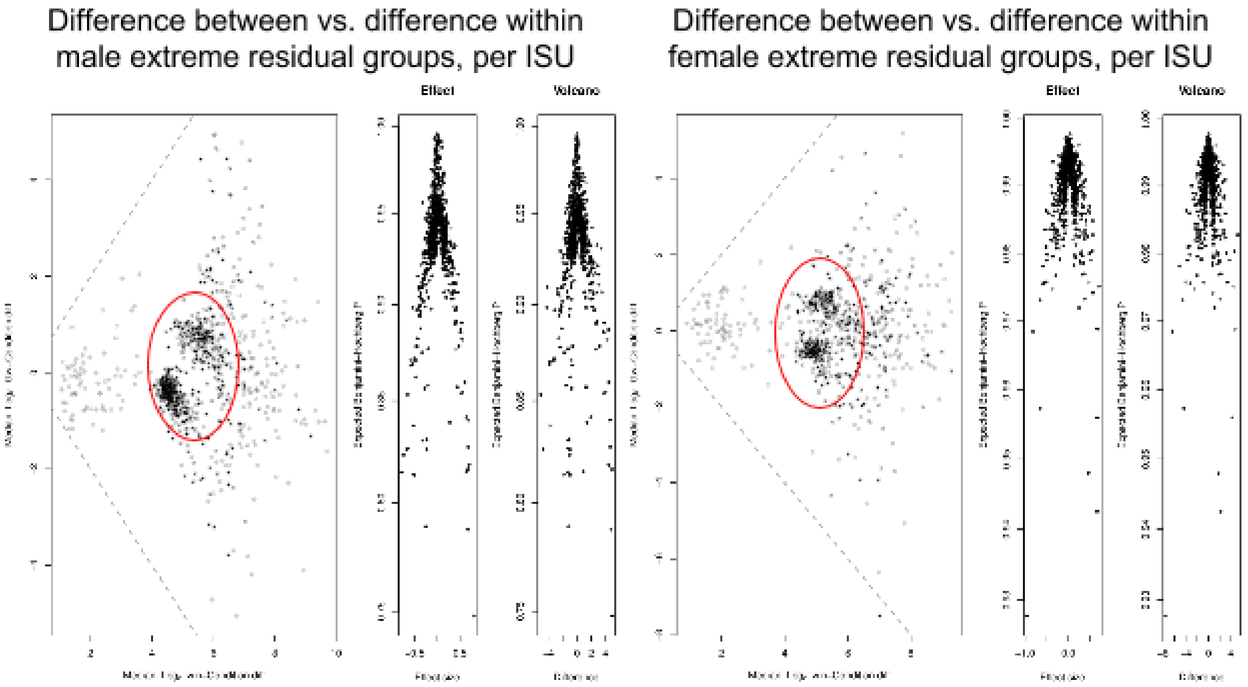

However, this effect disappears when the male and females are plotted together.

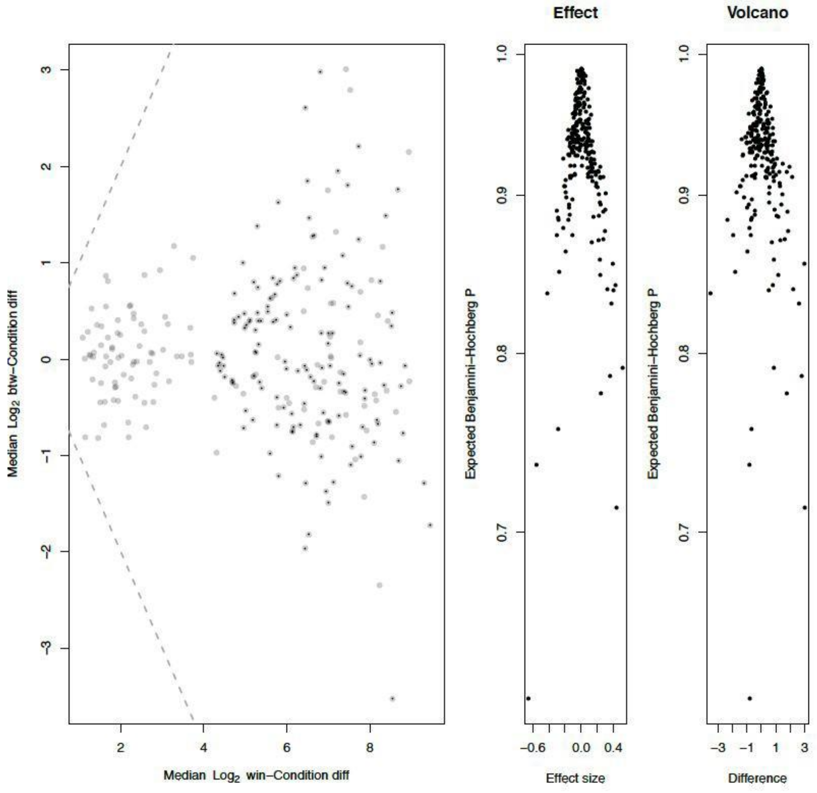

The difference within vs. difference between plot does not correspond between the female and male extreme residual groups.

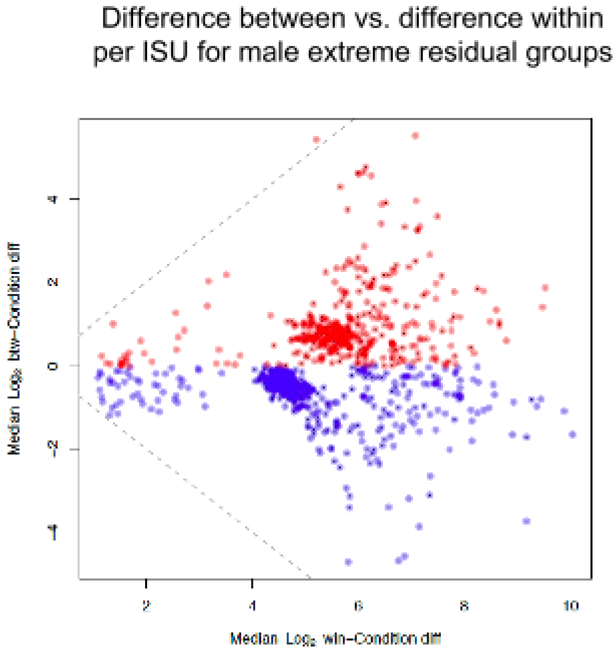

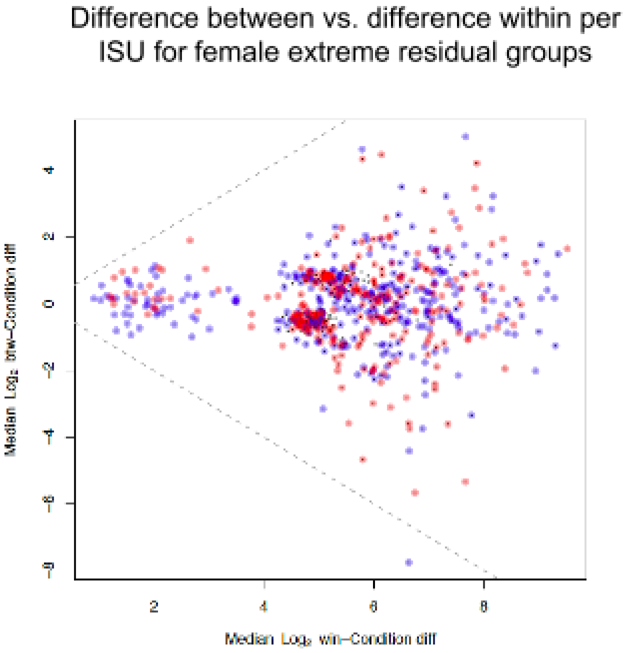

Additionally, if ALDEx2 is performed with only the rare OTUS (the OTUs with an abundance below the geometric mean), the effect sizes do not correspond between the female and male extreme residual groups.

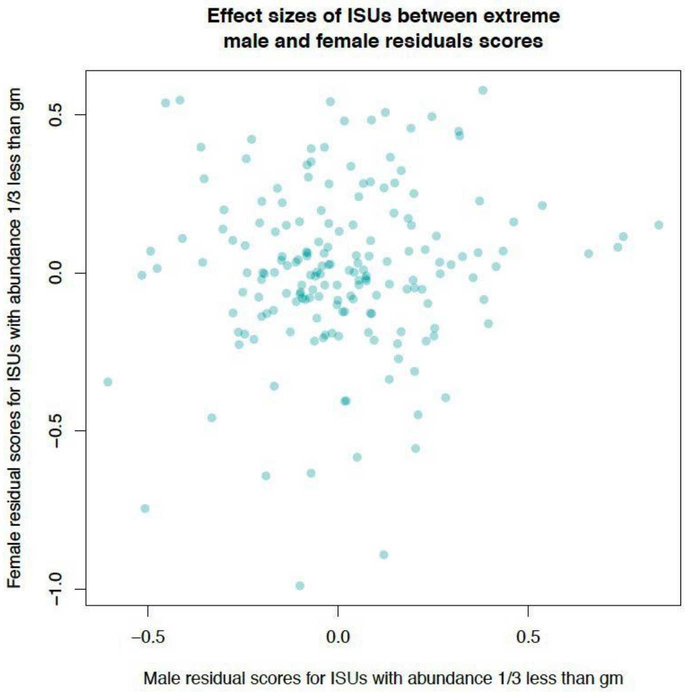

Lastly, we tried randomizing the samples in each condition, and found that the clusters always appeared, no matter which samples were in which groups. This appears to be an artifact of the DADA2 analysis.

Do the data show a consistent story?

There appears to be no correlation between the effect sizes of the male and female extreme residual groups, based on ALDEx analysis on both the OTU and ISU based approaches.

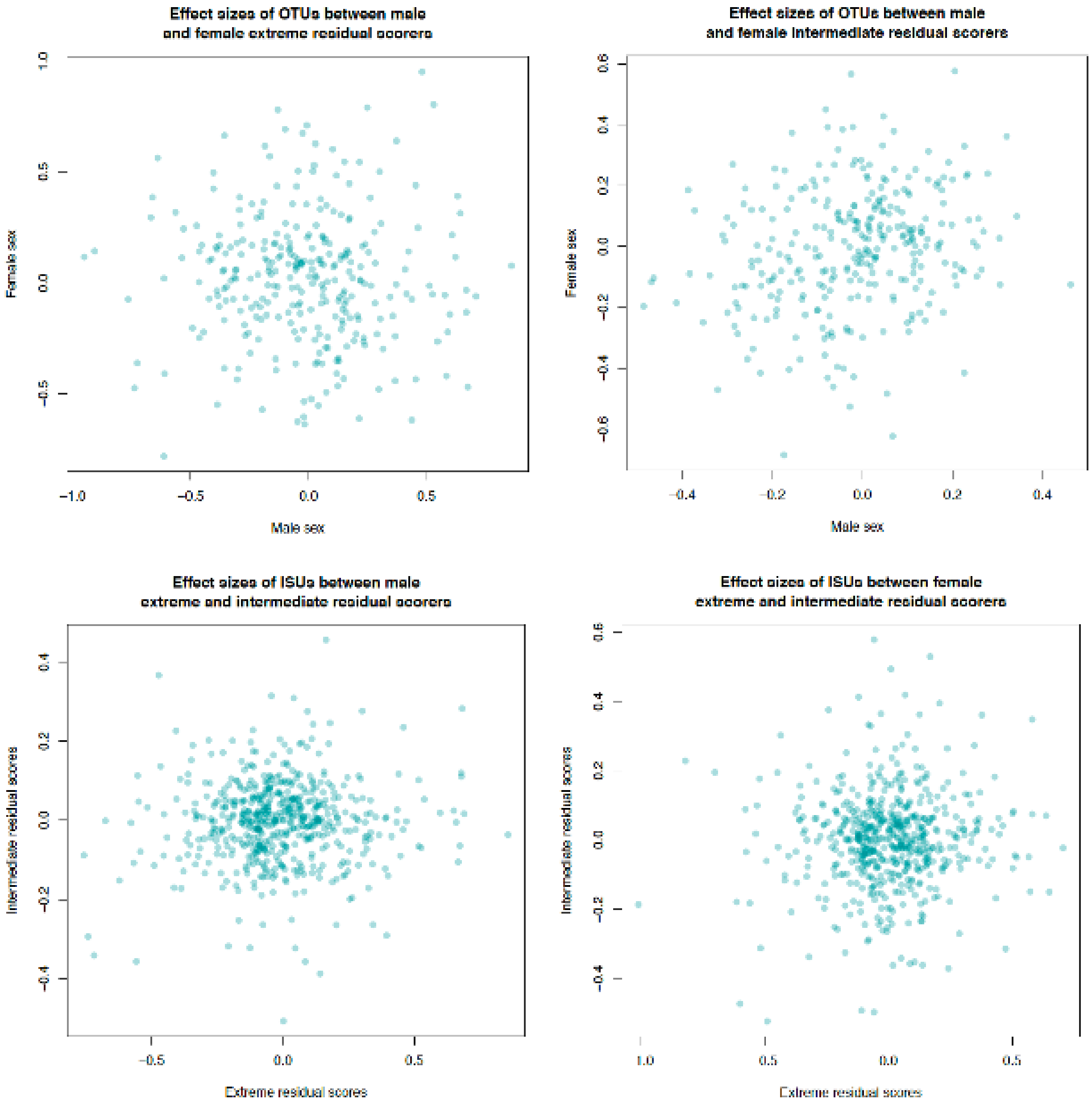

Additionally, no significant differences were seen between the male and female groups, when all patients of the male sex are compared with all patients of the female sex from this data set.

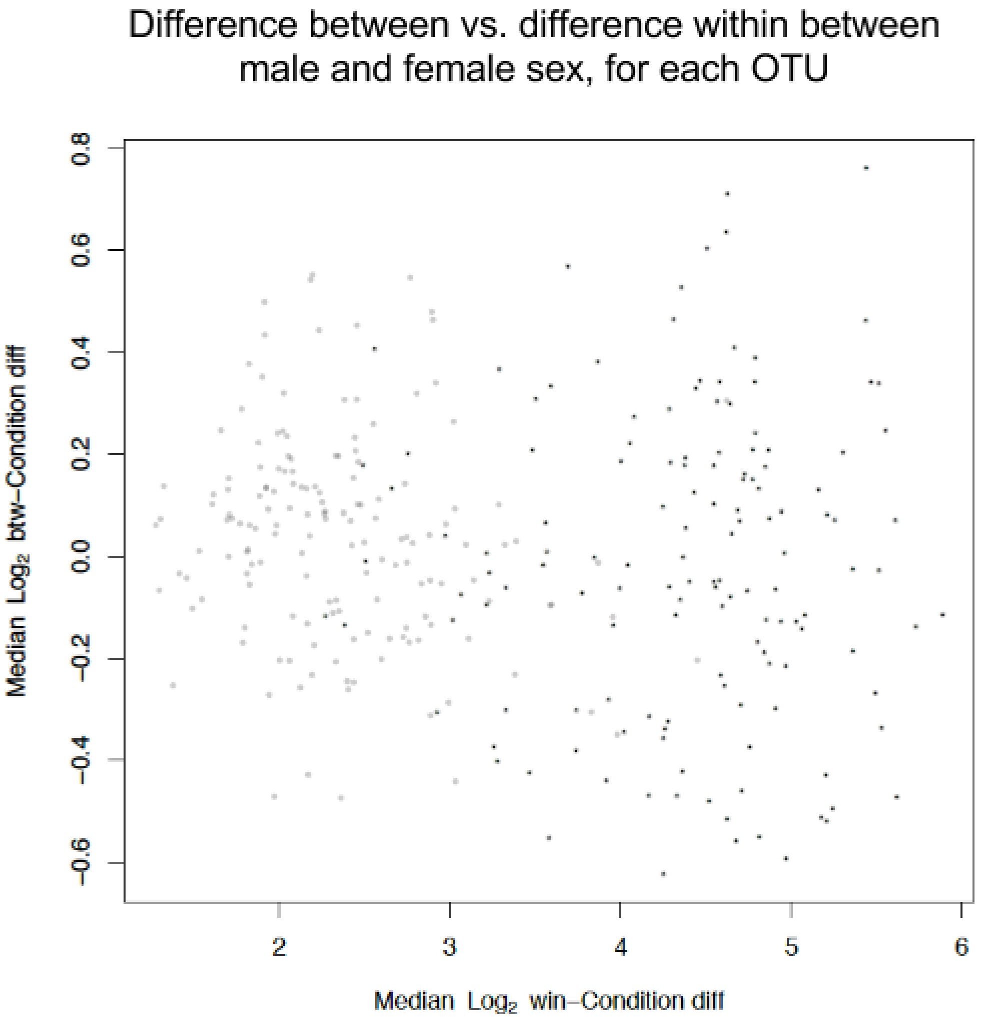

The effect sizes between the extreme (absolute residual > 2) and the intermediate groups (absolute residual between 1 and 2) also have no correlation in the OTU or the ISU based analysis.

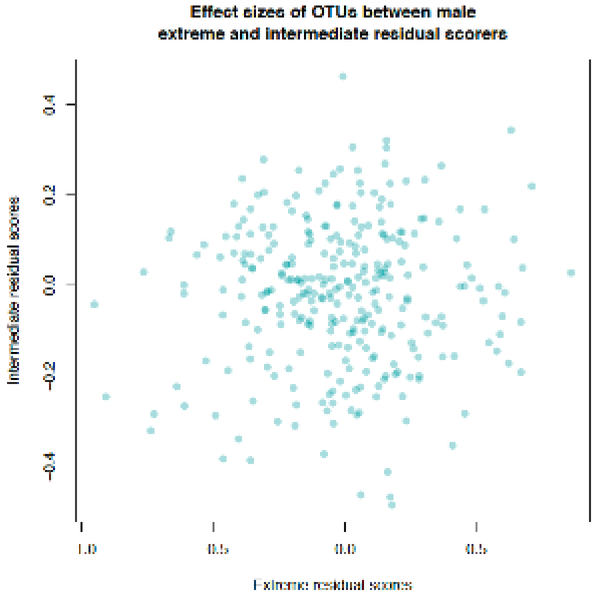

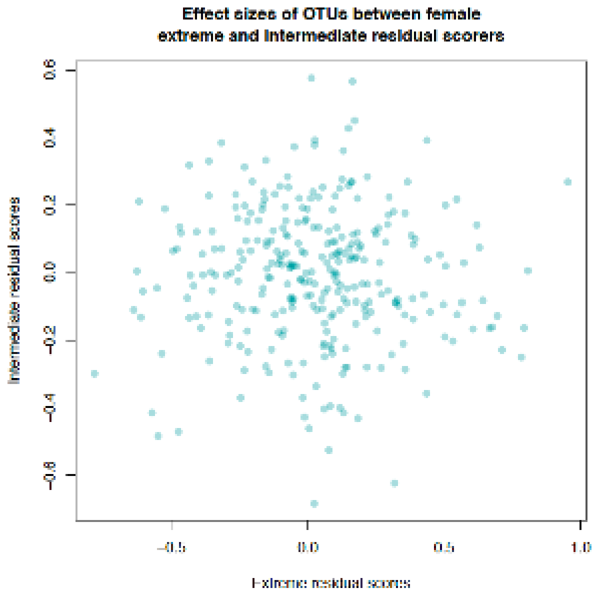

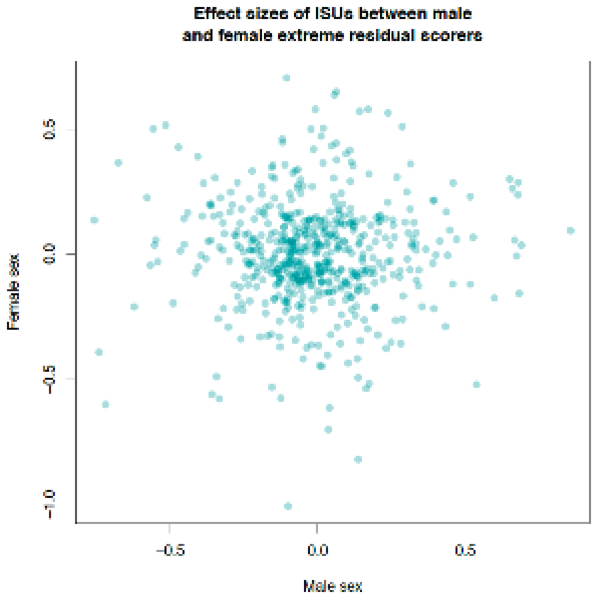

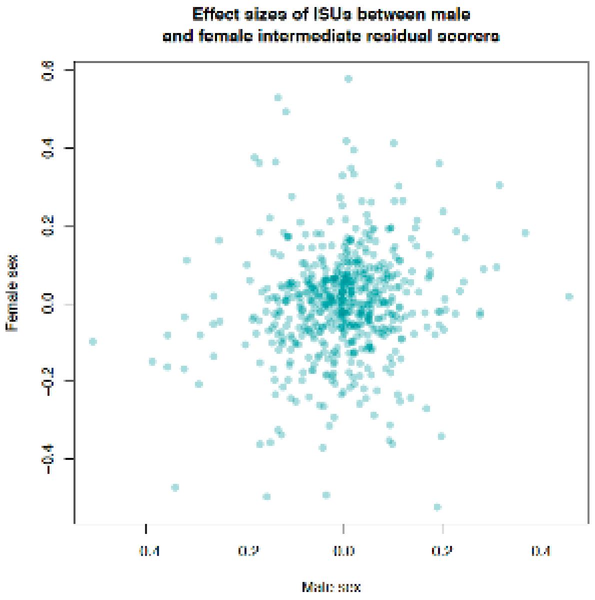

Is there any structure in the data?

Based on the biplots, there is no obvious structure in the data. These data have been aggregated at the genus level. The OTU and family level biplots are also available, as well as biplots derived from the ISU analysis.

Here is the biplot for the male patients with extreme residuals. The patients in the protected group are colored dark blue, and the patients in the unexplained group are colored dark red. The genus names are in grey. Note that the first two principal coordinates do not explain most of the variation in the data (18.4% and 12.5% respectively).

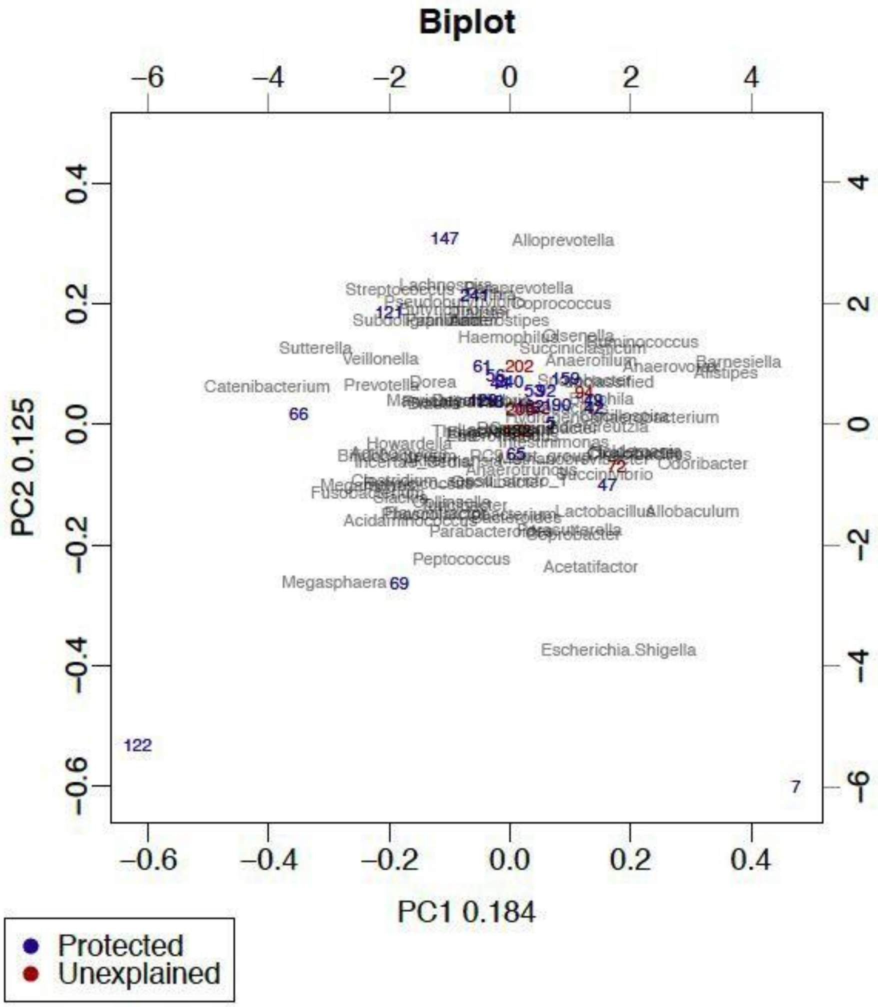

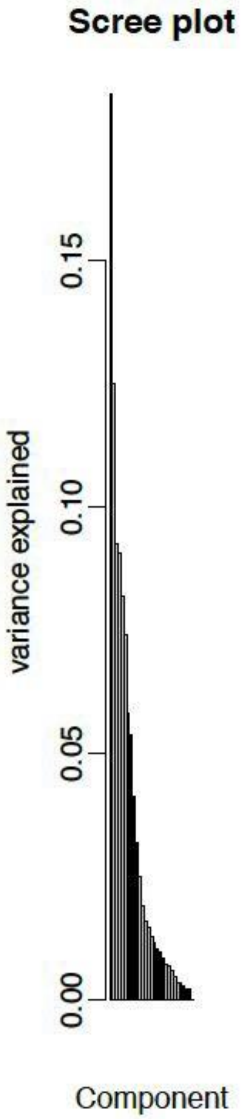

Here is the biplot for the female patients with extreme residuals. The patients in the protected group are colored dark blue, and the patients in the unexplained group are colored dark red. The genus names are in grey. Note that the first two principal coordinates do not explain most of the variation in the data (20.3% and 16.6% respectively).

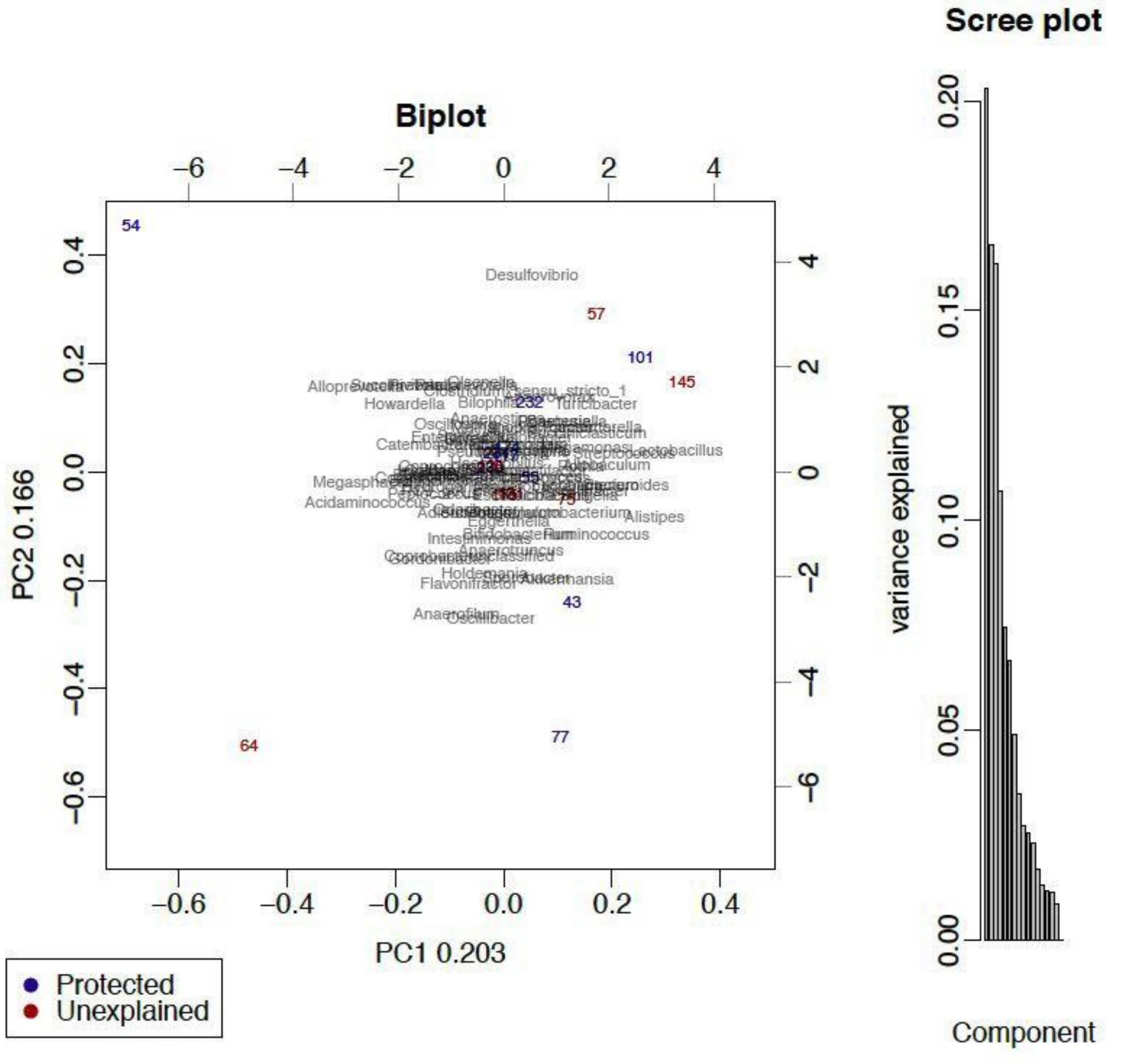

Here is the biplot for the male patients with intermediate residuals. The patients in the protected group are colored bright blue, and the patients in the unexplained group are colored bright red. The genus names are in grey. Note that the first two principal coordinates do not explain most of the variation in the data (11.7% and 10.9% respectively).

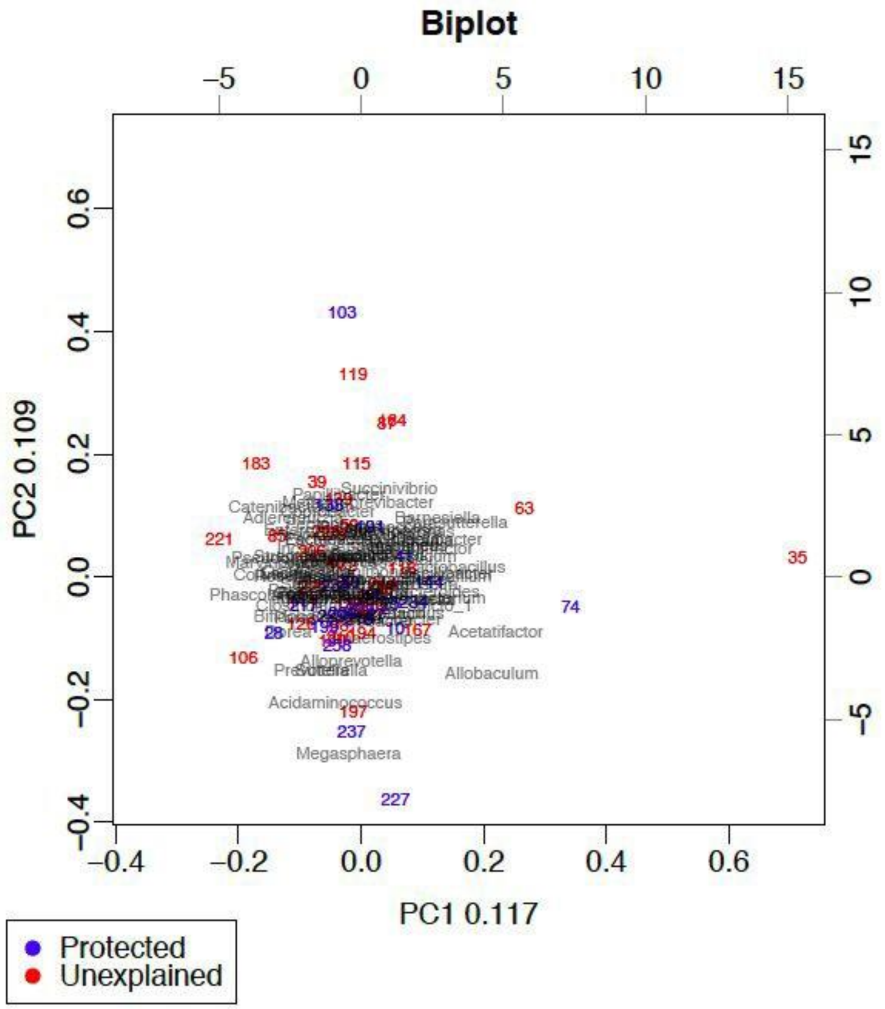

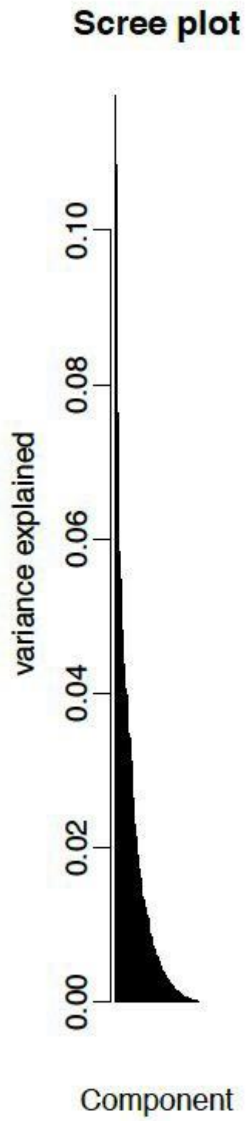

Here is the biplot for the female patients with intermediate residuals. The patients in the protected group are colored bright blue, and the patients in the unexplained group are colored bright red. The genus names are in grey. Note that the first two principal coordinates do not explain most of the variation in the data (12.6% and 10.3% respectively).

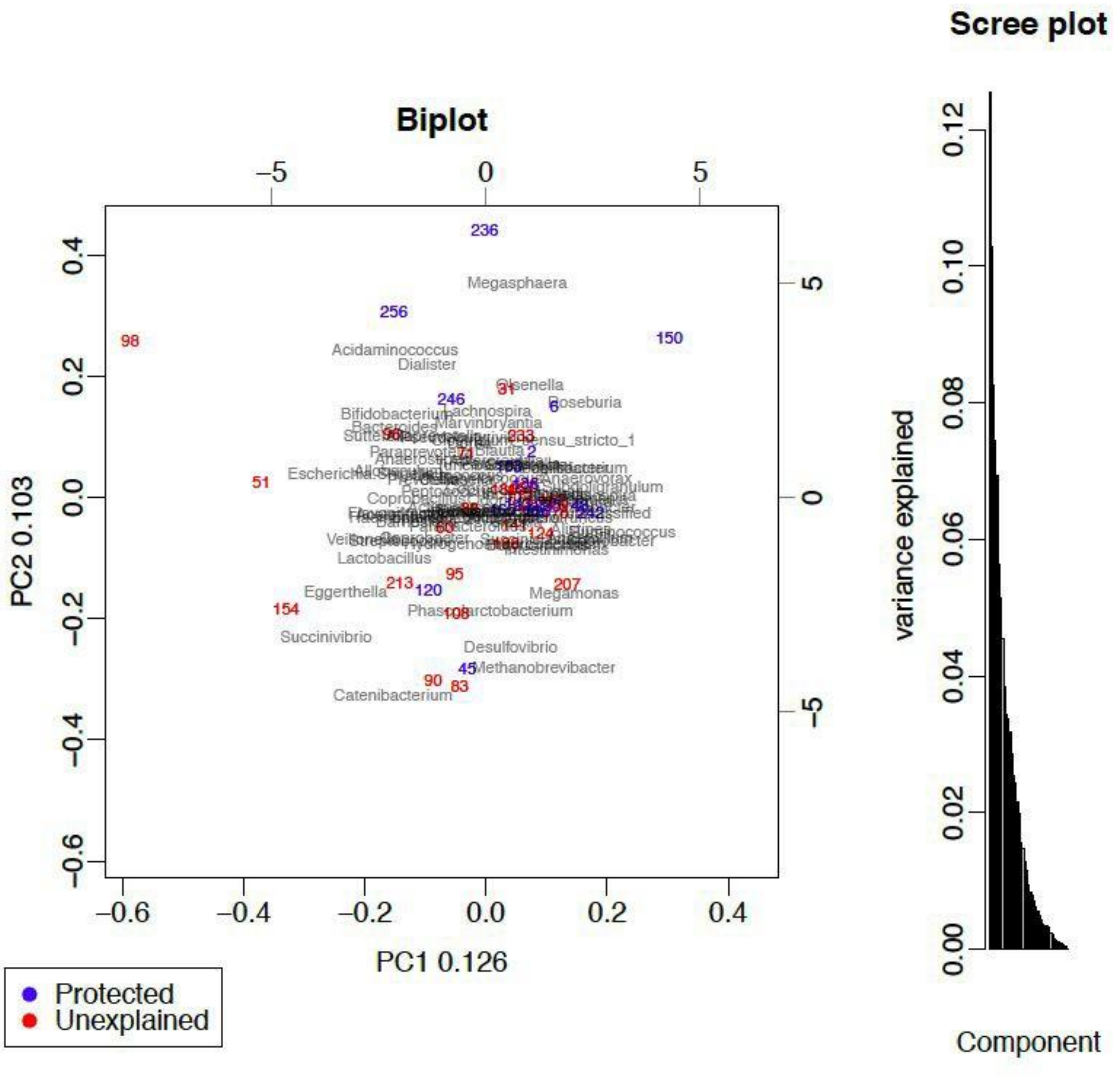

Principal components analysis have been performed with the extreme residual samples, as well as the top and bottom decile for each metadata. There is no obvious separation in any of the plots. Note the variance explained on each plot. These plots have all been performed at the OTU level.

Plots showing the levels of each type of metadata on the extreme residual samples are also available, but not shown here. Other metadata did not separate the extreme residual samples.

Below are PCoA plots performed on extreme residual samples (absolute residuals greater than or equal to 2).

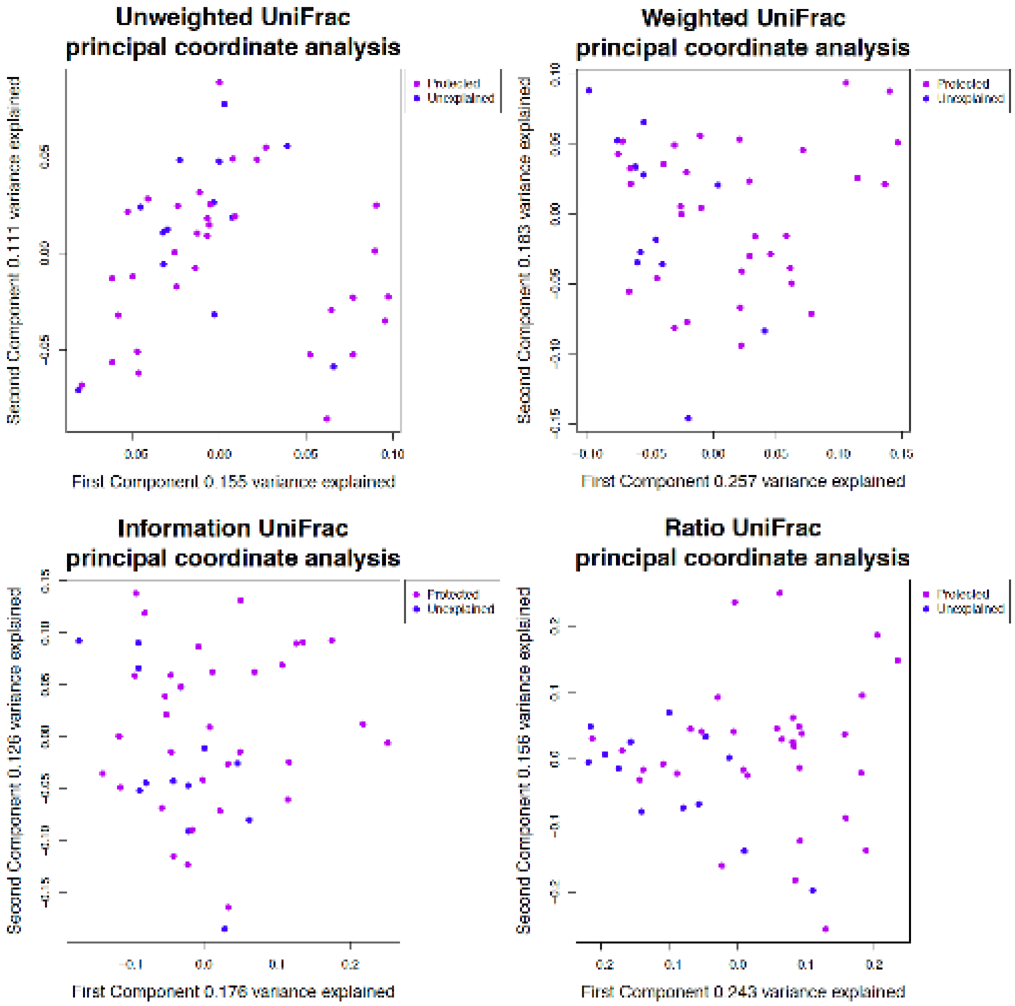

Below are PCoA plots performed on the samples with the top and bottom decile TPA.

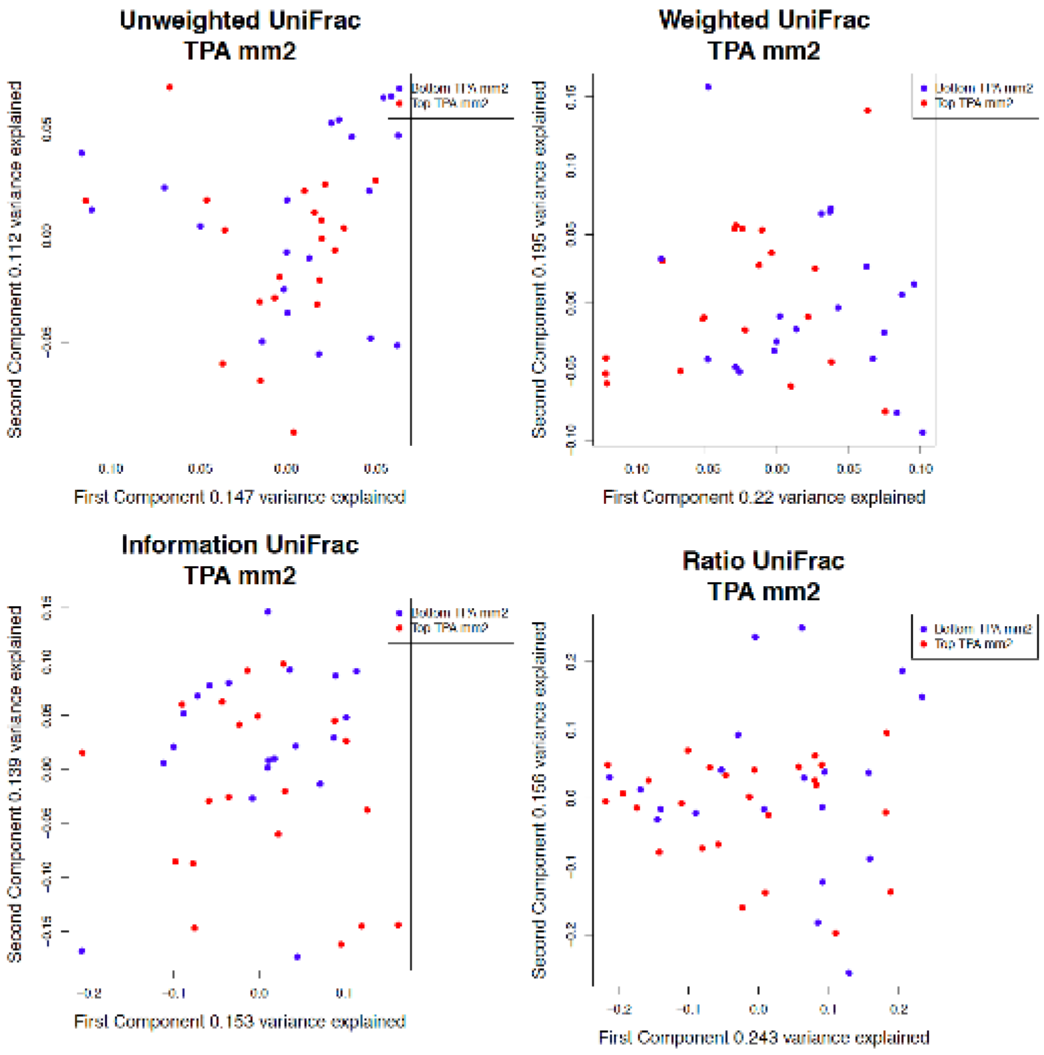

Below are PCoA plots performed on the samples with the top and bottom decile age.

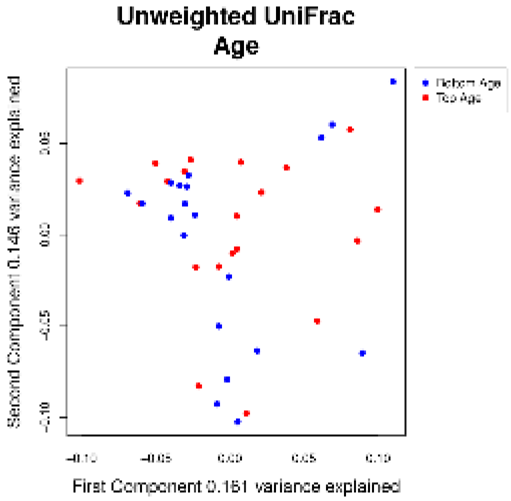

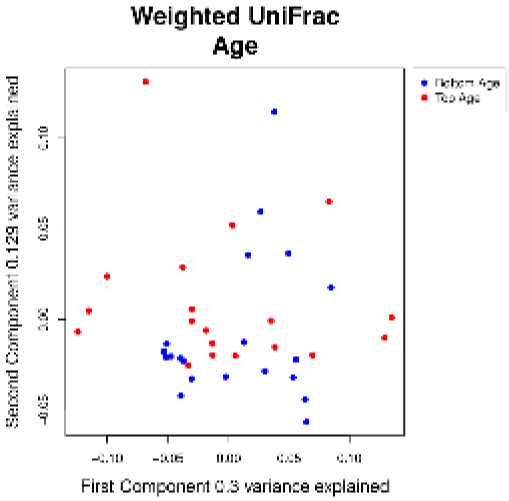

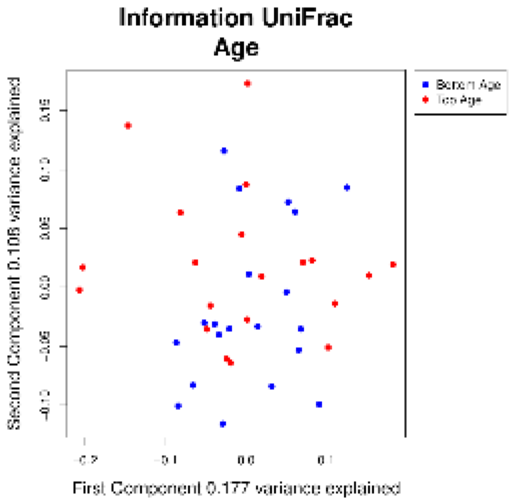

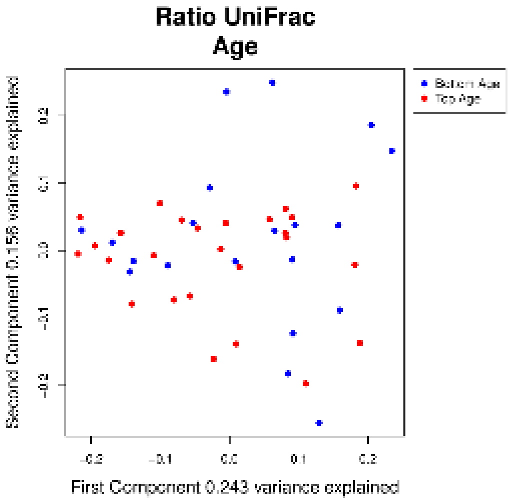

Below are PCoA plots performed on the samples with the top and bottom decile pack years.

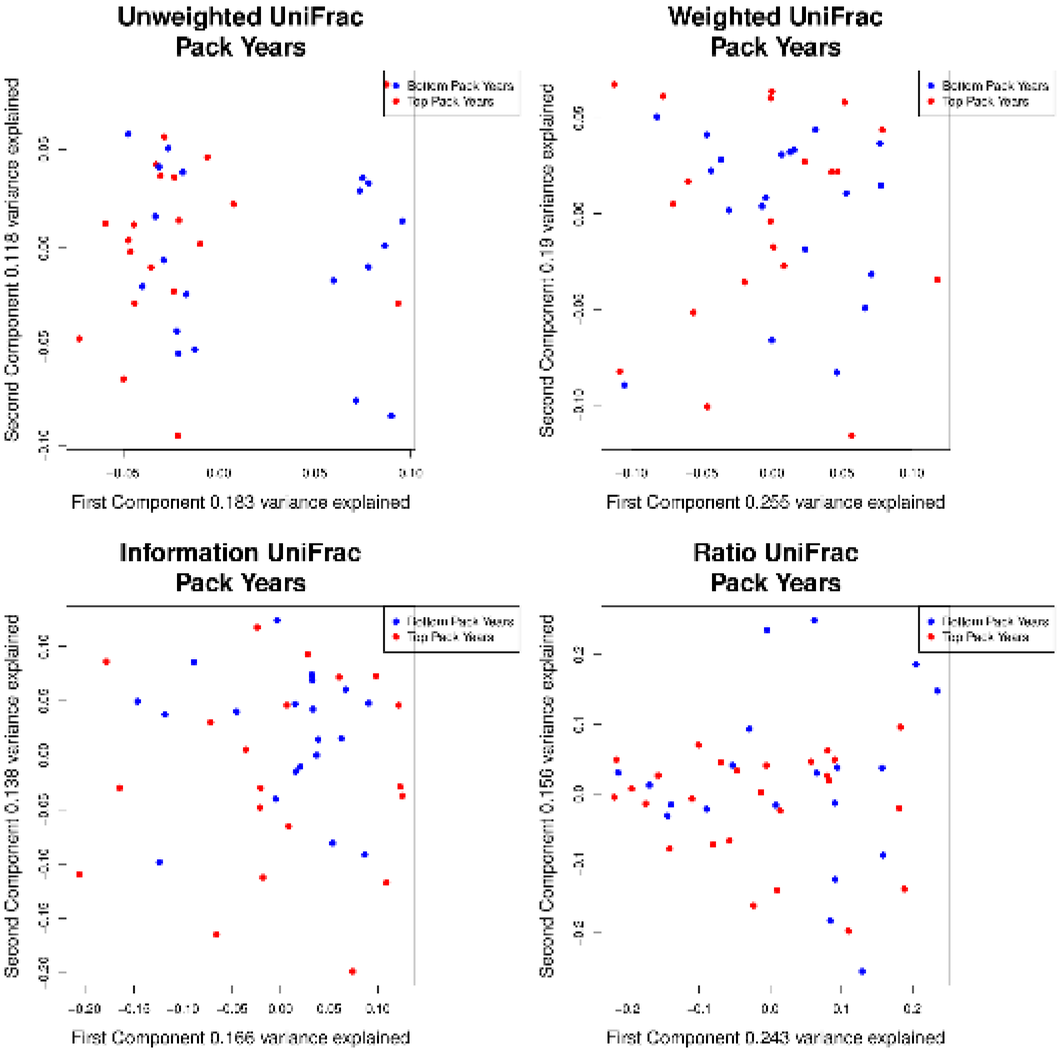

Below are PCoA plots performed on the samples with the top and bottom decile systolic blood pressure.

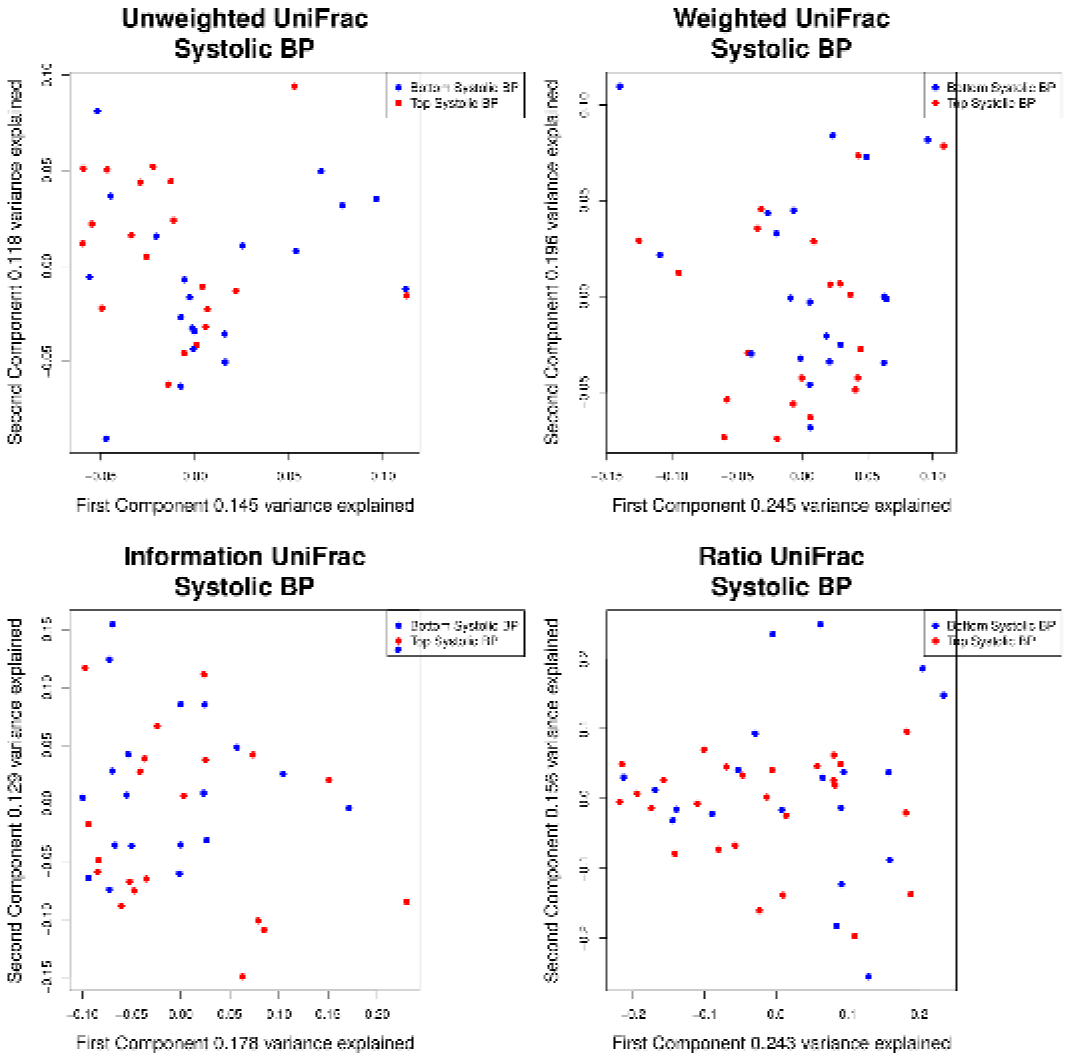

Below are PCoA plots performed on the samples with the top and bottom decile diastolic blood pressure.

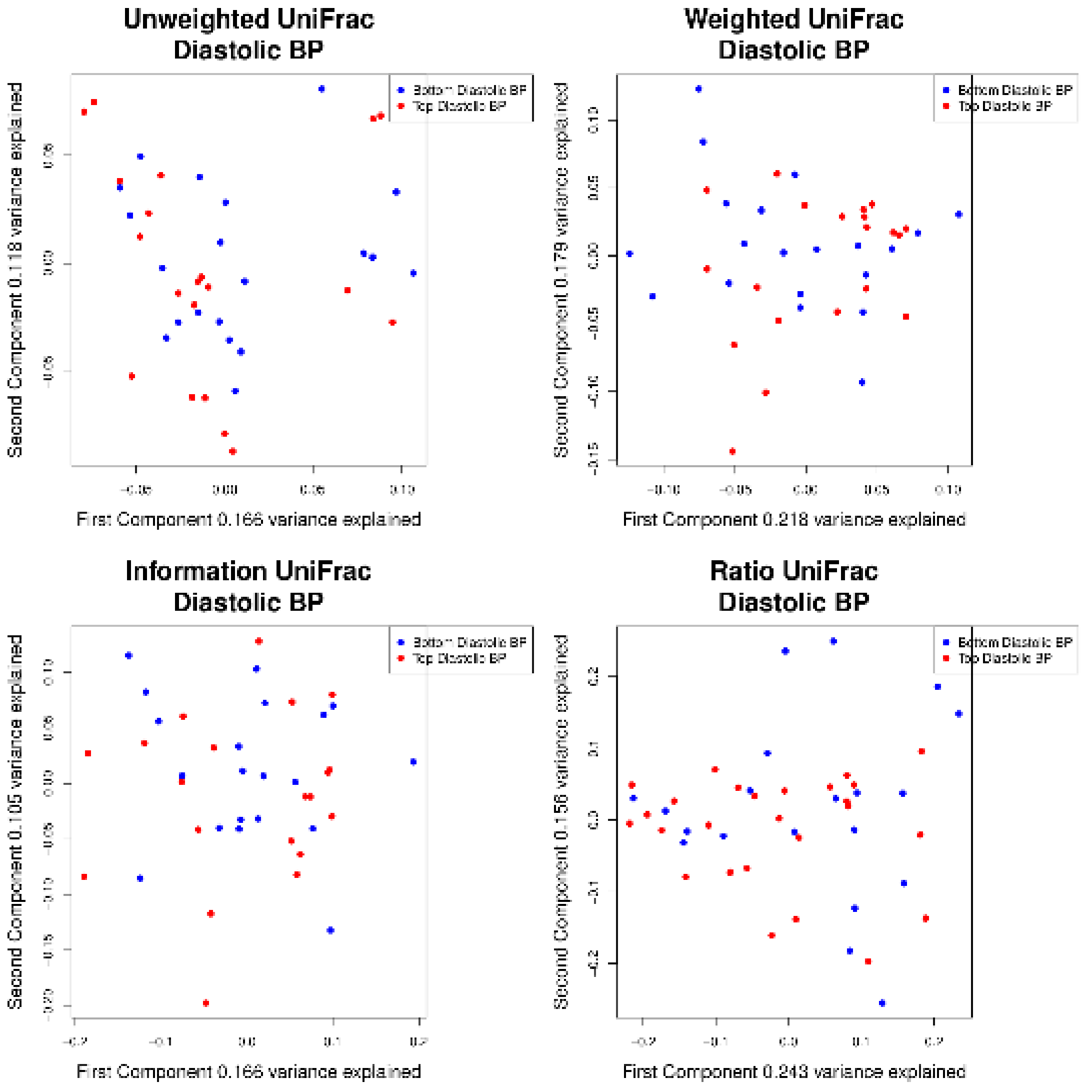

Below are PCoA plots performed on the samples with the top and bottom decile cholesterol levels.

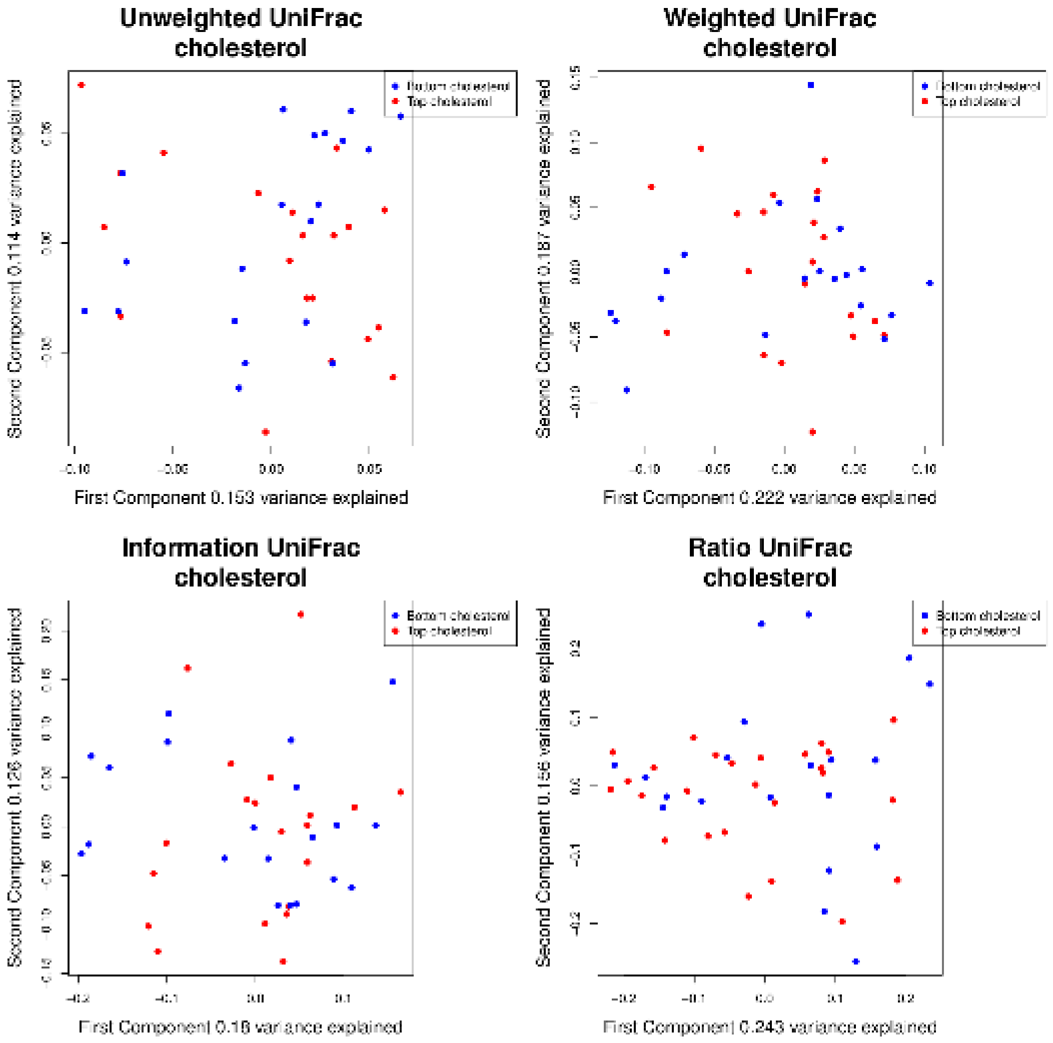

Below are PCoA plots performed on the samples with the top and bottom decile triglyceride levels.

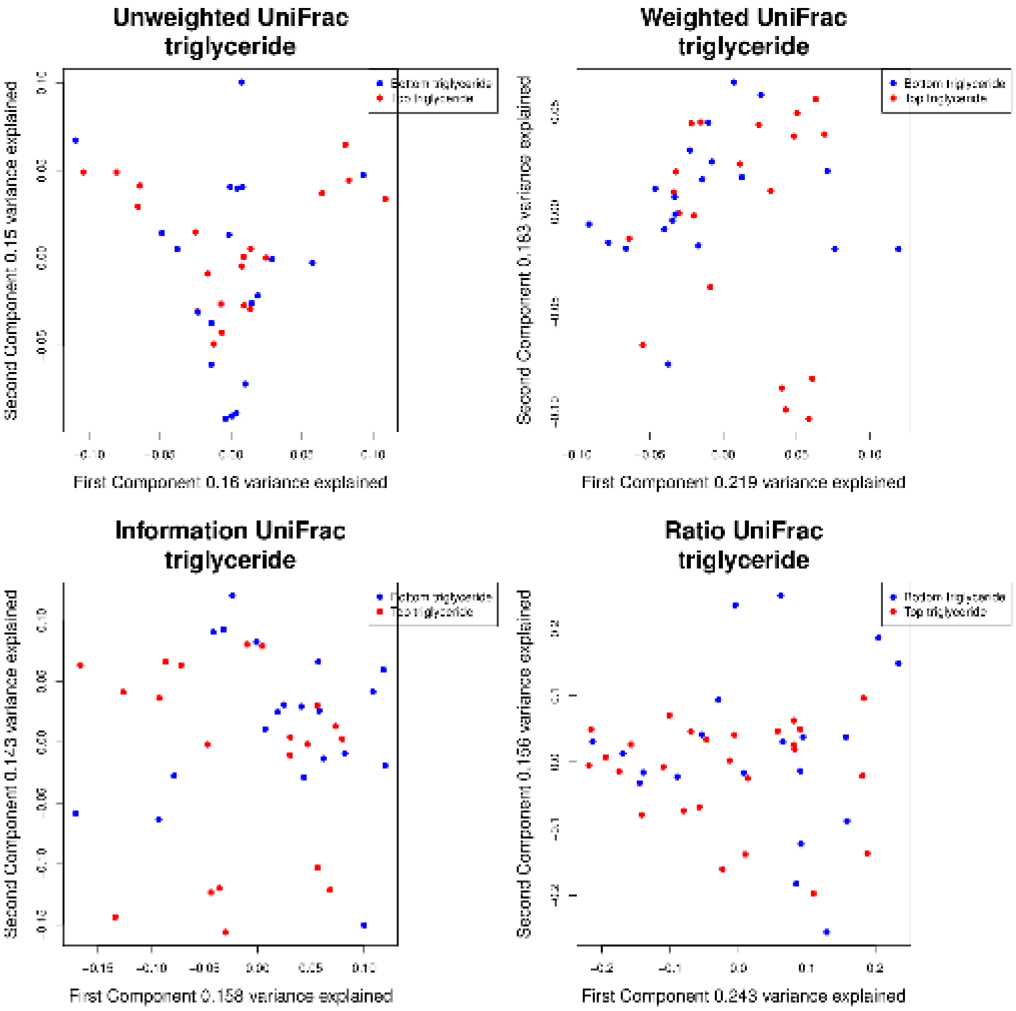

Below are PCoA plots performed on the samples with the top and bottom decile HDL levels.

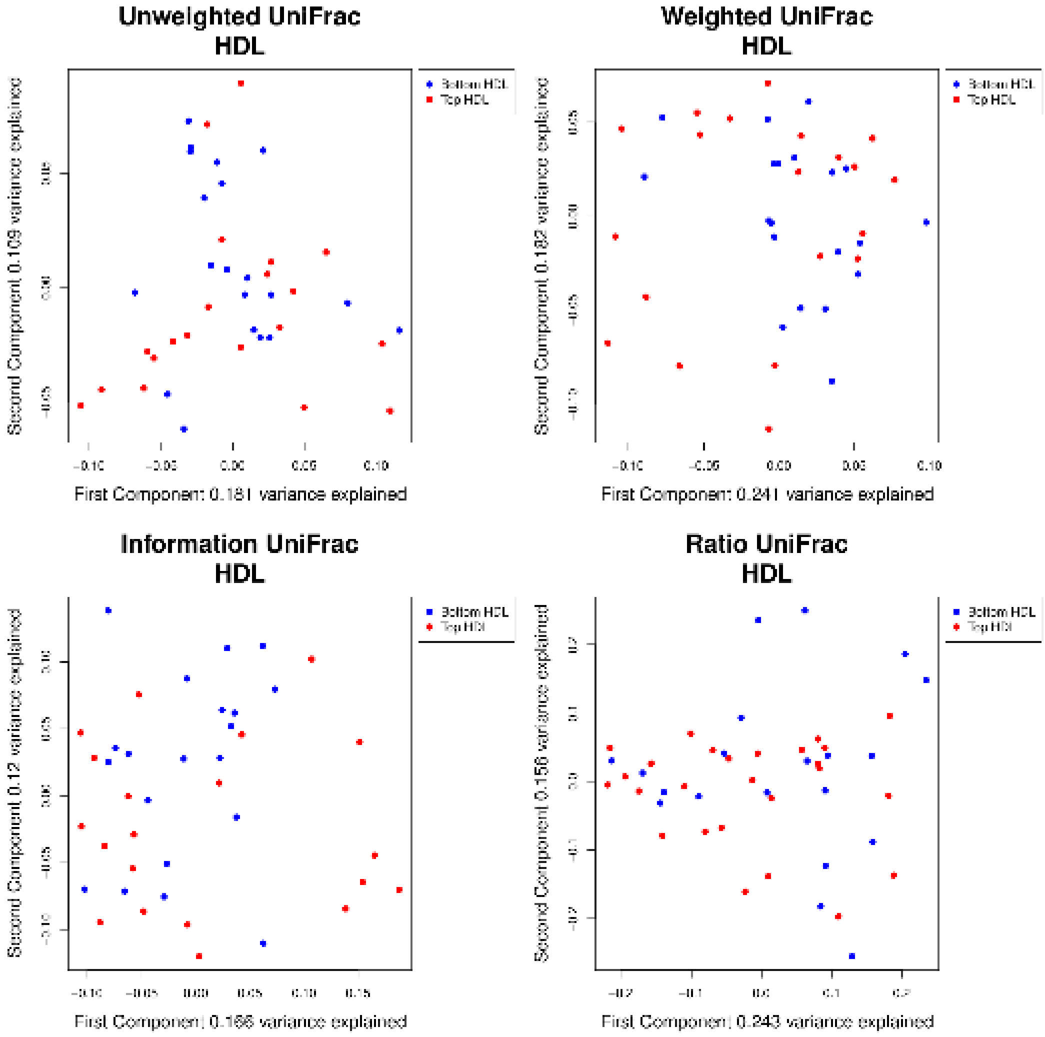

Below are PCoA plots performed on the samples with the top and bottom decile LDL levels.

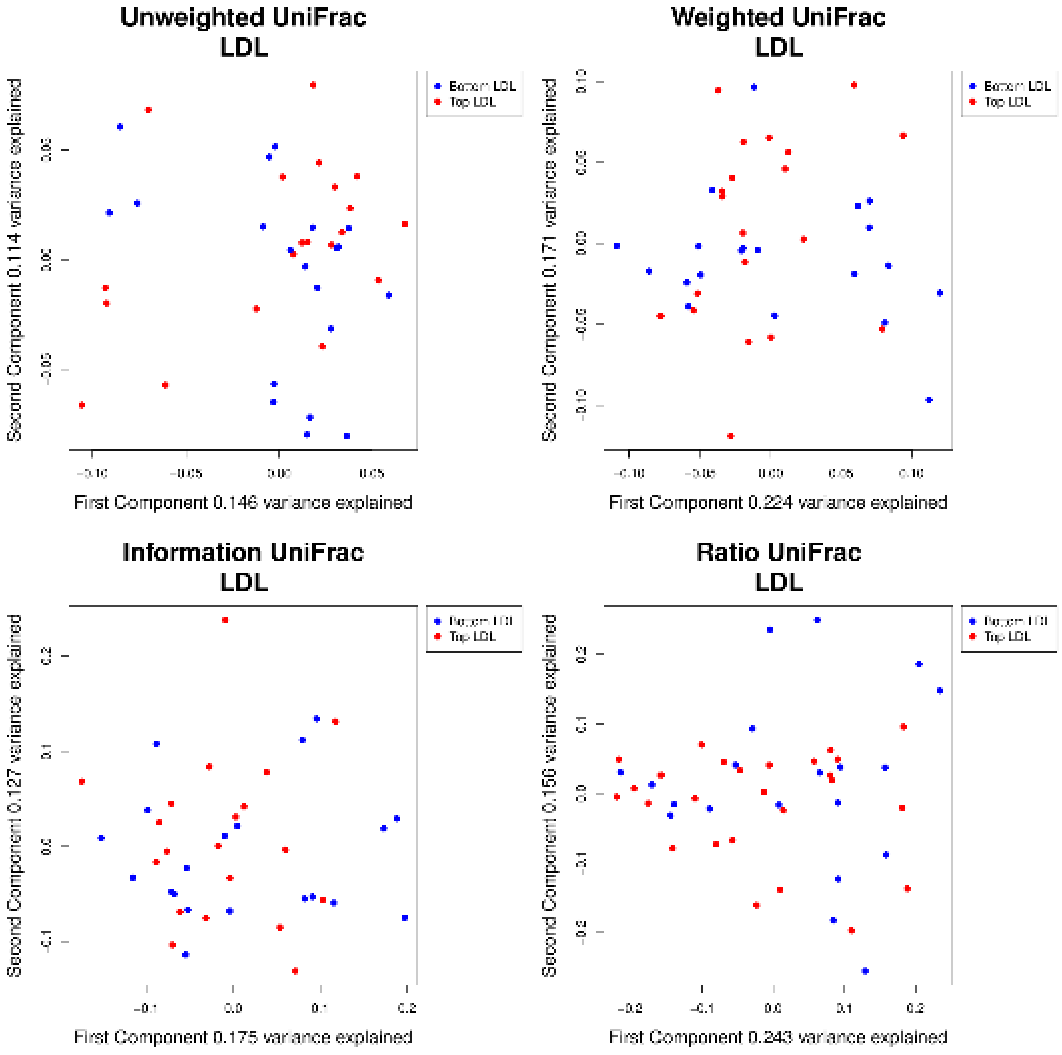

Below are PCoA plots performed on the samples with the top and bottom decile predicted standard values.

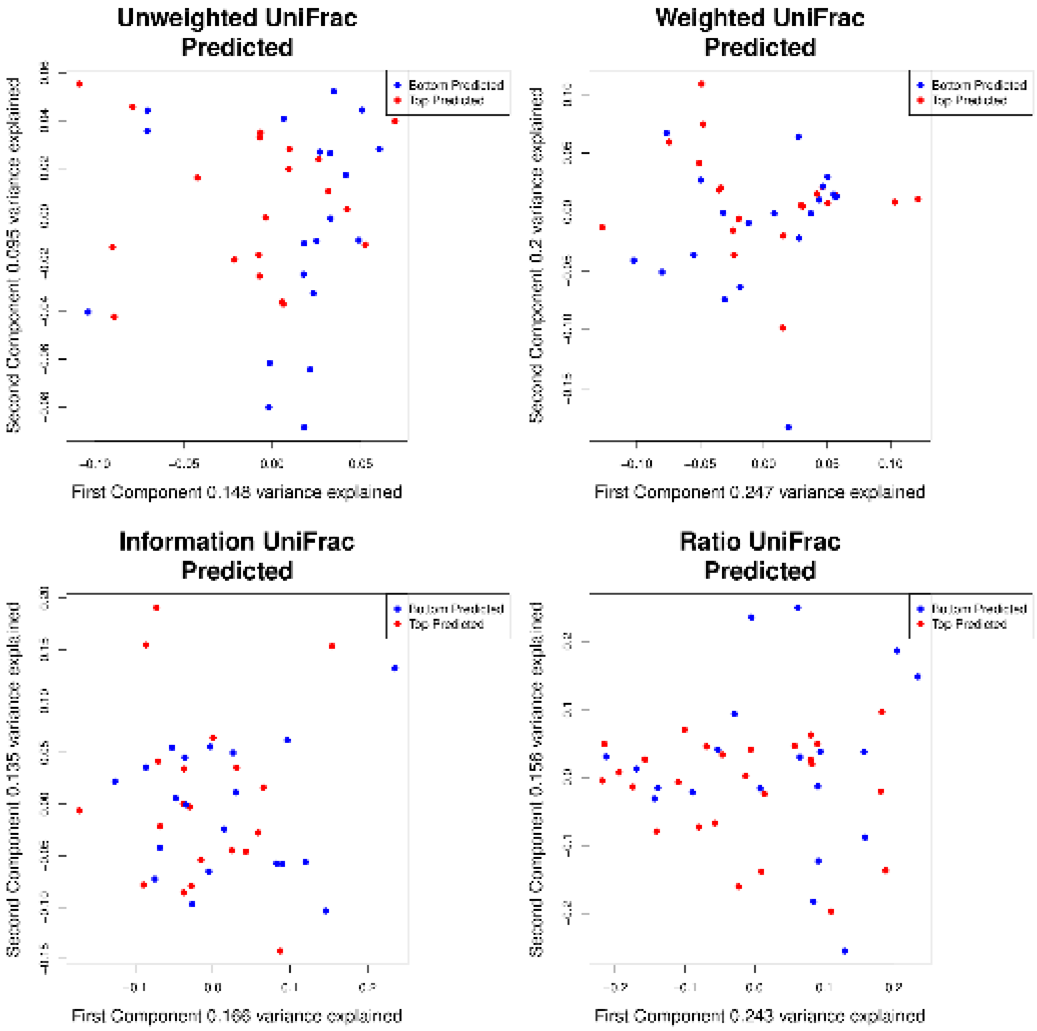

ALDEx was also performed on the male and female groups with extreme stenosis residual scores (absolute score greater than or equal to 2). No OTUs were found to be significantly differential between groups.

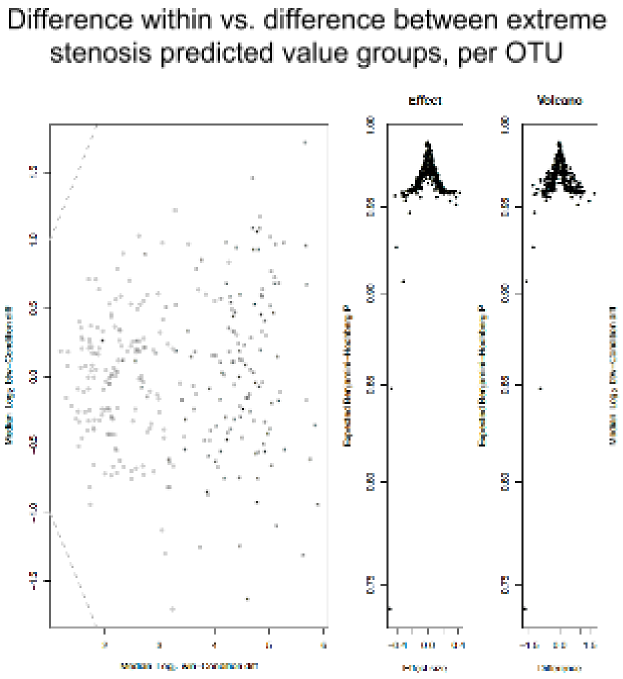

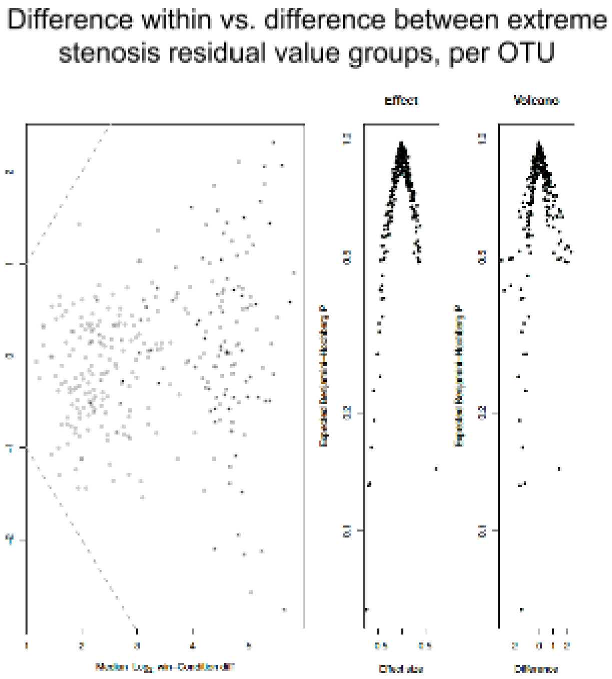

The effect sizes between TPA residual values and the stenosis residual values extreme group comparisons were not correlated. In this comparison, the samples present in both the extreme stenosis and TPA residual groups were removed.

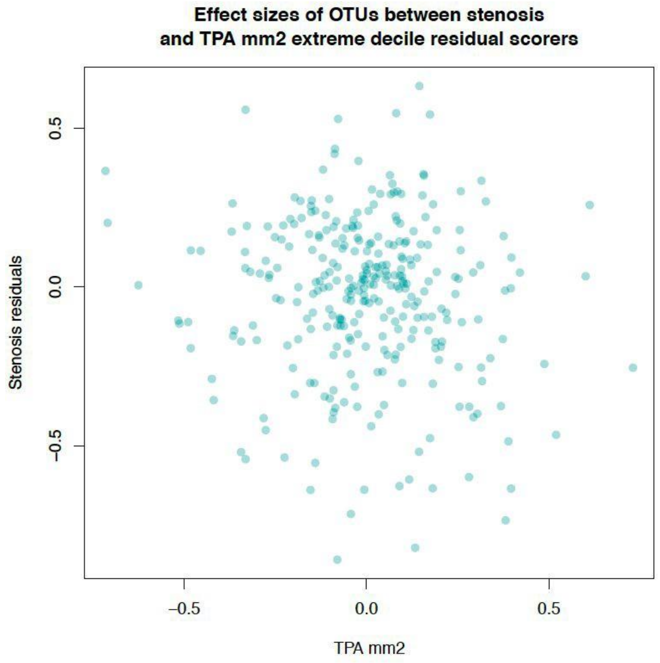

Supplement: Supplementary file 2 — Supplementary material [file mmc2.pdf]
